# Torsional vibration analysis of shaft with multi inertias

**DOI:** 10.1038/s41598-022-11211-x

**Published:** 2022-05-05

**Authors:** Tao Peng, Qun Yan

**Affiliations:** Aviation Science and Technology Key Laboratory of Aviation Acoustics and Vibration, Joint Laboratory of Turboprop Aircraft Vibration and Noise Reduction Technology, Aircraft Strength Research Institute of China, No. 86, Electronic 2nd Road, Yanta District, Xi’an, 710065 Shaanxi China

**Keywords:** Engineering, Mathematics and computing, Physics

## Abstract

An analytical method is proposed to investigate the torsional vibration of the uniform circular shaft with multiple concentrated inertias. The governing equation is established based on the Hamiltonian principle and verified by the dynamical method. The theoretical solutions of frequencies and mode shapes under different boundary conditions are obtained using the separation variable method and integral transformation. The effectiveness of the proposed method is verified by comparison with existing literature. Considering the change of the magnitudes/positions/number of concentrated inertias, and different boundary conditions, the natural frequencies and mode shapes are discussed. Several general rules are obtained. Moreover, some interesting phenomena have been found and explained. The analytical method has applications in the design of shafting with multiple concentrated inertias and the reliability checking of the “approximate” solutions.

## Introduction

Shafting is widely used in ships, naval vessels, rail coaches, aircraft, spacecraft, and other machinery. Torsional vibration of shafting is always a most important problem for engineers. Many engineering solutions have been proposed, such as the Rayleigh method^1,2^, lumped mass method (lumped parameter method, LMM), finite element method (FEM), and boundary element method (BEM)^3,4^. However, these mentioned methods are “approximate”, the “exact” theoretical solutions are rarely reported. In this paper, an analytical method based on the Hamilton principle, variable separation method and integral transformation is proposed to obtain the governing equation of torsional vibration of shafting with multi inertias and the “exact” theoretical solution.

Generally, a structural system may be considered as a “continuous” system or a “discrete” system according to the feasibility of the problem concerned^5^. One of the main differences between the mentioned two systems is the degree of freedom (DOF) of the system, which is infinite in the “continuous” system and finite in the “discrete” system. The solutions for the former are usually called “exact” (or closed-form) solutions and those for the latter are called the “approximate” solutions.

There are “exact” solutions for uniform continuous shafts without any lumped masses (rotary inertias, disks, or shaft couplings), springs (linear springs or rotational springs) or spring-mass systems, which have been given in any textbook on structural dynamics. Shafts carrying single mass/spring at special positions (the ends) are also studied^6^. However, shafts carrying any number of masses/springs at arbitrary positions are rarely reported. The reason is that obtaining the “exact” solutions for the shaft with a multi masses/springs system is difficult (the total DOF of the shaft is increased by masses/springs). The more mass/spring, the more difficult it is to establish and solve the governing equations. Moreover, “approximate” solutions are appropriate for studying complex structures and much easier to obtain the results via computers. But, the “exact” solutions are usually the benchmarks for evaluating the accuracy or checking the reliability of the “approximate” solutions. Therefore, it is still important to develop “exact” solutions.

To overcome the aforementioned difficulty, the concentrated elements model was proposed^7,8^. Thanks to the genius idea, the total DOF of the shaft is kept unchanged if the multi masses/springs system is equivalent to concentrated elements. Next, the primary problem is to obtain the natural frequencies and mode shapes^9–11^. Unfortunately, the core of most existing research has been focused on lateral vibration problems^1–5,12^, torsional vibration of the shaft with multiple concentrated elements is less addressed. The main reason is that for the same structure, the natural frequency of transverse vibration is lower than that of torsional vibration, which is more likely to cause structural vibration. However, for shafting, its main form of motion is torsion, so torsional vibration analysis is inevitable.

A few decades ago, the torsional vibration problem shown in the title was solved by using the Holzer method^13^. Then, the problem was tackled by the transfer matrix method (TMM), such as LMM, and FEM after the rapid development of computer technology^14,15^. Until 1975 (and 1979), Gorman^16^ (and Blevins^17^) presented a new method to obtain the “exact” expressions of the natural frequencies and mode shapes of a uniform shaft carrying single rotary inertia or torsional spring. In 1988, Rao^18^ gave the exact expressions for the torsional frequencies and mode shapes of the generally constrained shafts and piping with two disks. Reference^19^ studied the torsional vibration problem of a beam containing lumped elements under cantilever boundary conditions and fixed boundary conditions. As for the “exact” solutions of the torsional vibration of uniform shafts carrying multi concentrated elements, the information concerned is rare. Only Chen^20^ presented an “exact” solution for free torsional vibration of a uniform circular shaft carrying multiple concentrated elements adopting the numerical assembly method (NAM). But NAM^21–23^ does not give the “exact” expression of mode shapes, and the method needs to establish complex coefficient matrices, which is still quite complicated. In order to remove the barriers, an analytical method (AM) based on the Hamilton principle^6,24^, variable separation method, and integral transformation is used to perform the torsional vibration analysis of a uniform circular shaft carrying multiple concentrated elements (rotary inertias) with arbitrary magnitudes and locations.

The paper is organized as follows: In “Governing equations” section, the simplified model, the establishment and verification of the governing equation are introduced. “Solution of the torsional vibration equation” section gives the solution of the governing equation with different boundary conditions, including the analytical expressions of mode shapes natural frequencies. “Validation of the proposed method” section shows the validation of the proposed method compared with the existing literature. The applications of AM are presented in “Application of AM” section, and several special phenomena are discussed in “Discussion of some phenomena” section. Finally, in “Conclusion” section, the concluding remarks and applications are discussed.

## Governing equations

### Model of the problem

**Figure 1:**
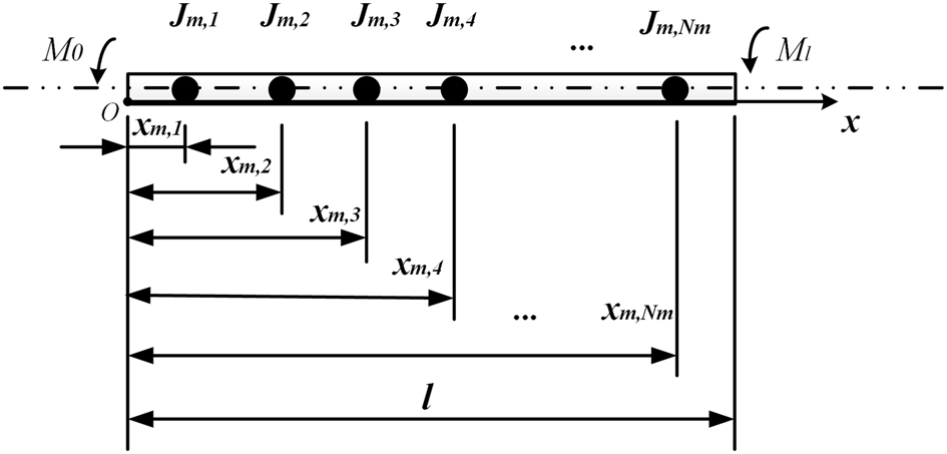
Physical model (This figure was created via Visio 2013, http://sj.njlhwlc.cn/pg/178.html, Figs. 2, 3, 4, 5, 12 and 21 were also created by Visio).

The physical model is shown in Fig. 1 by assuming the cross-section remains flat during torsion and the lateral deformation of the shaft is neglected. Therefore, only the torsional deformation of the shaft is considered.

Figure 1 shows a circular shaft carrying multi-concentrated elements. Where $$N_{m}$$: the number of the concentrated elements; $$l$$: the length of the shaft; $$D$$: the diameter of the shaft; $$x_{m,i}$$$$\left( {i = 1,2,3, \ldots ,N_{m} } \right)$$: the position of the $$i{\text{th}}$$ concentrated inertia; $$J_{m,i}$$: the value of the $$i{\text{th}}$$ concentrated inertia which is equivalent to the $$i{\text{th}}$$ disk with length $$H_{m,i}$$ and radius $$R_{m,i}$$, and $$J_{m,i} = {{\rho \pi R^{4}_{m,i} H_{m,i} } \mathord{\left/ {\vphantom {{\rho \pi R^{4}_{m,i} H_{m,i} } 2}} \right. \kern-\nulldelimiterspace} 2}$$; $$M_{0}$$: torque at $$x = 0$$; $$M_{l}$$: torque at $$x = l$$; $$\rho$$: the density of shaft and disks; $$G$$: shear modulus; $$\delta (x)$$: Dirac function.

### Governing equations established by the Hamiltonian principle

The angular displacement of each cross-section is $\theta = \theta (x,t)$ and $\gamma = r{{\partial \theta } \mathord{\left/ {\vphantom {{\partial \theta } {\partial x}}} \right. \kern-\nulldelimiterspace} {\partial x}}$ is the shear strain at each point in the cross-section. Hence, considering the constitutive relation, the shear stress at each point in the cross-section is $\tau = G\gamma$.

The deformation energy of the shaft can be expressed as

1 $$U = \frac{1}{2}\int_{0}^{l} {GI_{p} } (x)\left( {\frac{\partial \theta }{{\partial x}}} \right)^{2} dx,$$

where

2 $$I_{P} (x) = \int_{0}^{r(x)} {\int_{0}^{2\pi } {r^{3} } } d\varphi dr.$$

$I_{P} (x)$ represents the moment of inertia of the cross-section. Considering the problem of uniform cross-section shaft $I_{P} (x)$ is constant which degenerates into $I_{P}$.

The kinetic energy of the shaft obeys

3 $$T = \frac{1}{2}\int_{0}^{l} {\int_{0}^{r(x)} {\int_{0}^{2\pi } {\rho \left( {\frac{\partial \theta }{{\partial t}}} \right)^{2} } } } \theta r^{3} d\varphi drdx + \frac{1}{2}\sum\limits_{i = 1}^{{N_{m} }} {\int_{0}^{l} {J_{m,i} } \left( {\frac{\partial \theta }{{\partial t}}} \right)^{2} \delta (x - x_{m,i} )dx} .$$

The work of torque has the form of

4 $$W = M_{0} \left. \theta \right|_{x = 0} { + }M_{l} \left. \theta \right|_{x = l} .$$

The principle of Hamilton for non-conservative systems is written as

5 $$\delta \int_{{t_{0} }}^{{t_{1} }} {(T - U + W)} dt = \int_{{t_{0} }}^{{t_{1} }} {\delta Tdt - \int_{{t_{0} }}^{{t_{1} }} {\delta Udt} + \int_{{t_{0} }}^{{t_{1} }} W } dt = 0.$$

Substituting Eqs. (1)–(4) into Eq. (5) and considering $\left. {\delta \theta } \right|_{{t = t_{0} }} = 0$ and $\left. {\delta \theta } \right|_{{t = t_{1} }} = 0$, energy variation is shown as

6 $$\begin{aligned} \delta \int_{{t_{0} }}^{{t_{1} }} {\left( {T - U + W} \right)dt} = & \int_{{t_{0} }}^{{t_{1} }} {\int_{0}^{l} {\left( { - \rho I_{p} \frac{{\partial^{2} \theta }}{\partial t} - \sum\limits_{i = 1}^{{N_{m} }} {J_{m,i} \delta \left( {x - x_{m,i} } \right)\frac{{\partial^{2} \theta }}{\partial t} + GI_{p} \frac{{\partial^{2} \theta }}{\partial x}} } \right)} } \delta \theta dxdt \\ & + \int_{{t_{0} }}^{{t_{1} }} {\left( {\left. {M_{0} - GI_{p} \frac{\partial \theta }{{\partial x}}} \right|_{x = 0} } \right)} \left. {\delta \theta } \right|_{x = l} dt + \delta \int_{{t_{0} }}^{{t_{1} }} {\left( {\left. {M_{l} + GI_{p} \frac{\partial \theta }{{\partial x}}} \right|_{x = l} } \right)} \left. {\partial \theta } \right|_{x = 0} dt. \\ \end{aligned}$$

Equation (6) is suitable for arbitrary $t_{0}$, $t_{1}$. Then, $\delta \theta ,\left. {\delta \theta } \right|_{x = l} \left. {,\delta \theta } \right|_{x = 0}$ are arbitrary. Thus, the vibration differential equation can be expressed as

7 $$\rho I_{P} \frac{{\partial^{2} \theta }}{{\partial t^{2} }}{ + }\sum\limits_{i = 1}^{{N_{m} }} {J_{m,i} \delta (x - x_{m,i} )} \frac{{\partial^{2} \theta }}{{\partial t^{2} }} - GI_{P} \frac{{\partial^{2} \theta }}{{\partial x^{2} }} = 0.$$

The boundary conditions are written as

8 $$M_{0} + GI_{p} \left. {\frac{\partial \theta }{{\partial x}}} \right|_{x = 0} = 0,\;\;\left. {M_{l} - GI_{p} \frac{\partial \theta }{{\partial x}}} \right|_{x = l} = 0.$$

When the concentrated inertia is not taken into account, the equation is simplified into the differential equation of free vibration of the continuous beam

9 $$\rho I_{P} \frac{{\partial^{2} \theta }}{{\partial t^{2} }} - GI_{P} \frac{{\partial^{2} \theta }}{{\partial x^{2} }} = 0.$$

Considering free-boundary conditions (free-free, FF), Eq. (11) can be expressed as

10 $$\left. {\frac{\partial \theta }{{\partial x}}} \right|_{x = 0,l} = 0.$$

When the torque is zero and the boundary are fixed (pinned–pinned, PP),

11 $$\left. \theta \right|_{x = 0,l} = 0.$$

Supposing $M_{0} = M_{l} = 0$, the mixed boundary condition (free–pinned, FP) is given as

12 $$\left. {\frac{\partial \theta }{{\partial x}}} \right|_{x = 0} = 0,\;\;\left. \theta \right|_{x = l} = 0.$$

Or (pinned–free, FP)

13 $$\left. \theta \right|_{x = 0} = 0,\;\;\left. {\frac{\partial \theta }{{\partial x}}} \right|_{x = l} = 0.$$

### The verification of governing equations

**Figure 2:**
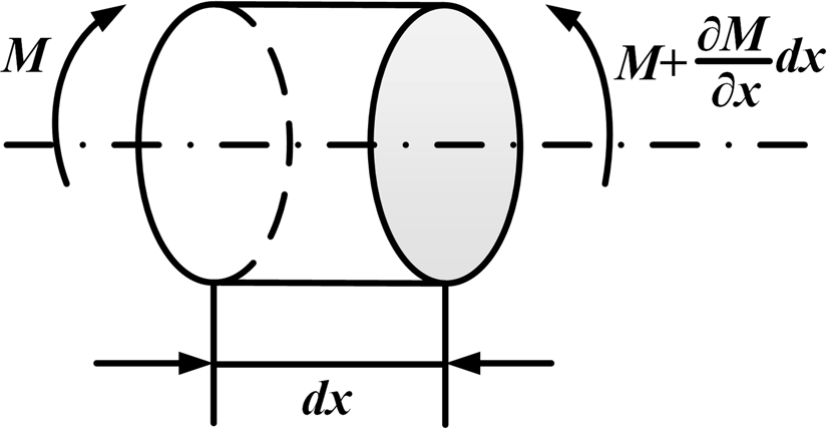
Micro-segment without concentrated elements.

To verify the correctness of the vibration equation established in “Governing equations established by the Hamiltonian principle” section, it is analyzed from the perspective of torque balance. Figure 2 shows a beam element without concentrated elements (rotary inertias).

**Figure 3:**
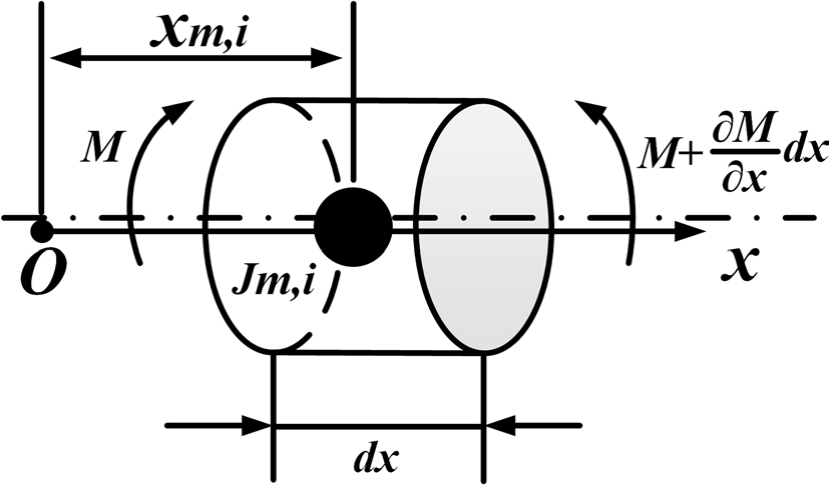
Micro-segment with concentrated elements.

The length of the beam element is $dx$, the torque on its left is $M$ and on the right side is $M + {{\partial M} \mathord{\left/ {\vphantom {{\partial M} {\partial x}}} \right. \kern-\nulldelimiterspace} {\partial x}}dx$. Figure 3 shows a beam element with the $i$-th concentrated rotary inertia located at $x = x_{m,i}$, and has the same geometric parameters as Fig. 2.

When the micro-segment shown in Fig. 2 does not contain the concentrated rotary inertia, the equilibrium equation can be obtained

14 $$\rho I_{P} dx\frac{{\partial^{2} \theta }}{{\partial t^{2} }} - \left( {M + \frac{\partial M}{{\partial x}}dx - M} \right) = 0.$$

When the micro-section contains the $i$-th concentrated inertia, Eq. (12) becomes into

15 $$\rho I_{P} \frac{{\partial^{2} \theta }}{{\partial t^{2} }}{ + }J_{m,i} \delta (x - x_{m,i} )\frac{{\partial^{2} \theta }}{{\partial t^{2} }} - \frac{\partial M}{{\partial x}} = 0.$$

Supposing multiple concentrated inertias, the equation expression obeys

16 $$\rho I_{P} \frac{{\partial^{2} \theta }}{{\partial t^{2} }}{ + }\sum\limits_{i = 1}^{{N_{m} }} {J_{m,i} \delta (x - x_{m,i} )} \frac{{\partial^{2} \theta }}{{\partial t^{2} }} - \frac{\partial M}{{\partial x}} = 0,$$

where

17 $$M = GI_{P} \frac{\partial \theta }{{\partial x}}.$$

Substituting Eq. (17) into Eq. (16), the differential equation of vibration is the same as Eq. (7). In the same way, the boundary conditions can be obtained, which are coincident with Eqs. (8), (10)–(13). Therefore, the theory applied in this paper is credible, and the equations established are also available.

## Solution of the torsional vibration equation

### Frequencies and mode shapes for different boundary conditions

Applying the method of separating variables

18 $$\theta (x,t) = \theta (x)T(t).$$

Make $\mathop T\limits^{..} (t) = \frac{{\partial^{2} T(t)}}{{\partial t^{2} }}$ and $\theta^{\prime\prime}(x) = \frac{{\partial^{2} \theta (x)}}{{\partial x^{2} }}$, substitute Eq. (18) into Eq. (7), thus

19 $$\left[ {\rho I_{P} + \sum\limits_{i = 1}^{{N_{m} }} {J_{m,i} \delta (x - x_{m,i} )} } \right]\theta (x)\mathop T\limits^{..} (t) - GI_{P} \theta^{\prime\prime}(x)T(t) = 0.$$

Suppose

20 $$\frac{{GI_{P} \theta^{\prime\prime}(x)}}{{\left[ {\rho I_{P} + \sum\limits_{i = 1}^{{N_{m} }} {J_{m,i} \delta (x - x_{m,i} )} } \right]\theta (x)}} = \frac{{\mathop T\limits^{..} (t)}}{T(t)} = - \lambda ,\;\left( {\lambda > 0} \right).$$

Then

21 $$\mathop T\limits^{..} (t) + \lambda T(t) = 0,$$

22 $$GI_{P} \theta^{^{\prime\prime}} (x) + \lambda \left[ {\rho I_{P} + \sum\limits_{i = 1}^{{N_{m} }} {J_{m,i} \delta (x - x_{m,i} )} } \right]\theta (x) = 0.$$

Equation (22) is the equation of mode shape, which can be simplified using Laplace transform as

23 $$Y(s) = \frac{s}{{s^{2} + \lambda {\raise0.7ex\hbox{$\rho $} \!\mathord{\left/ {\vphantom {\rho G}}\right.\kern-\nulldelimiterspace} \!\lower0.7ex\hbox{$G$}}}}\theta (0) - \frac{1}{{s^{2} + \lambda {\raise0.7ex\hbox{$\rho $} \!\mathord{\left/ {\vphantom {\rho G}}\right.\kern-\nulldelimiterspace} \!\lower0.7ex\hbox{$G$}}}}\theta^{^{\prime}} (0) - \sum\limits_{i = 1}^{{N_{m} }} {\frac{{J_{m,i} }}{{GI_{p} }}} \frac{\lambda }{{s^{2} + \lambda {\raise0.7ex\hbox{$\rho $} \!\mathord{\left/ {\vphantom {\rho G}}\right.\kern-\nulldelimiterspace} \!\lower0.7ex\hbox{$G$}}}}\theta (x_{m,\;i} )e^{{ - x_{m,\;i} s}} ,$$

where $Y(s) = \int_{0}^{ + \infty } {\theta \left( x \right)} e^{ - sx} dx$, $s$ is the complex parameter; $\theta (0)$, $\theta^{\prime}(0)$ are the displacement and the velocity at $x = 0$ separately, $\theta^{\prime}(x) = \frac{\partial \theta (x)}{{\partial x}}$.

Make $\omega = \sqrt {\lambda {\rho \mathord{\left/ {\vphantom {\rho G}} \right. \kern-\nulldelimiterspace} G}}$, the equation of mode shapes can be obtained by inverse Laplace transform:

24 $$\theta (x) = \theta (0)\cos \omega x + \frac{{\theta^{\prime}(0)}}{\omega }\sin \omega x - \frac{\omega }{{\rho I_{p} }}\sum\limits_{i = 1}^{{N_{m} }} {J_{m,i} \theta (x_{m,i} )} \sin \omega (x - x_{m,i} )H(x - x_{m,i} ).$$

$\theta (x_{m,i} ) = \theta (0)\cos \omega x_{m,i} + \frac{{\theta^{\prime}(0)}}{\omega }\sin \omega x_{m,i} - \frac{\omega }{{\rho I_{p} }}\sum\limits_{j = 1}^{i} {J_{m,j} \theta (x_{m,j} )} \sin \omega (x_{m,j} - x_{m,i} )H(x_{m,j} - x_{m,i} )$, and $H(x)$ is the unit step function,

### Free boundary shaft (free–free, FF)

Applying Eq. (10), the eigenfunction is:

25 $$\omega \left[ {\sin \omega l + \frac{\omega }{{\rho I_{p} }}\sum\limits_{i = 1}^{{N_{m} }} {J_{m,i} \left( {\cos \omega x_{m,i} - \frac{\omega }{{\rho I_{p} }}\sum\limits_{j = 1}^{i - 1} {\frac{{J_{m,j} \theta (x_{m,j} )}}{\theta (0)}\sin \omega \left( {x_{m,i} - x_{m,j} } \right)} } \right)\cos \omega \left( {l - x_{m,i} } \right)} } \right] = 0.$$

Mode shape is

26 $$\theta (x) = \theta (0)\cos \omega x - \frac{\omega }{{\rho I_{p} }}\sum\limits_{i = 1}^{{N_{m} }} {J_{m,i} \theta (x_{m,i} )\sin \omega \left( {x - x_{m,i} } \right)} H\left( {x - x_{m,i} } \right).$$

### Fixed boundary shaft (pinned–pinned, PP)

Applying Eq. (11), the eigenfunction is:

27 $$\frac{\sin \omega l}{\omega } - \frac{\omega }{{\rho I_{p} }}\sum\limits_{i = 1}^{{N_{m} }} {J_{m,i} \left( {\frac{{\sin \omega x_{m,i} }}{\omega } - \frac{\omega }{{\rho I_{p} }}\sum\limits_{j = 1}^{i - 1} {\frac{{J_{m,j} \theta (x_{m,j} )}}{{\theta^{^{\prime}} (0)}}\sin \omega (x_{m,j} - x_{m,i} )} } \right)\sin \omega (l - x_{m,i} )} = 0.$$

Mode shape is

28 $$\theta (x) = \theta^{^{\prime}} (0)\frac{sin\omega x}{\omega } - \frac{\omega }{{\rho I_{p} }}\sum\limits_{i = 1}^{{N_{m} }} {J_{m,i} \theta (x_{m,i} )\sin \omega \left( {x - x_{m,i} } \right)} H\left( {x - x_{m,i} } \right).$$

### Mixed boundary shaft (pinned–free, FP)

Applying Eqs. (12) or (13), the eigenfunction is:

29 $$\cos \omega l - \frac{\omega }{{\rho I_{p} }}\sum\limits_{i = 1}^{{N_{m} }} {J_{m,i} \left( {\cos \omega x_{m,i} - \frac{\omega }{{\rho I_{p} }}\sum\limits_{j = 1}^{i - 1} {\frac{{J_{m,j} \theta (x_{m,j} )}}{\theta (0)}\sin \omega \left( {x_{m,i} - x_{m,j} } \right)} } \right)\sin \omega (l - x_{m,i} )} = 0.$$

Mode shape is

30 $$\theta (x) = \theta (0)\cos \omega x - \frac{\omega }{{\rho I_{p} }}\sum\limits_{i = 1}^{{N_{m} }} {J_{m,i} \theta (x_{m,i} )\sin \omega \left( {x - x_{m,i} } \right)} H\left( {x - x_{m,i} } \right).$$

## Validation of the proposed method

In order to validate the proposed method, three cases were studied and compared with the results published in Refs.^6,16,20^.

### Case one

**Figure 4:**
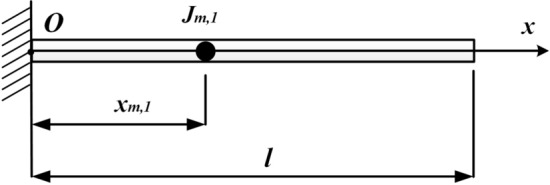
The cantilevered shaft carrying single rotary inertia.

The cantilevered shaft carrying single rotary inertia, located at $x_{{m,1}} /l = 1.0$ or 0.5 respectively, were studied first (Fig. 4).

The dimensions and physical properties of the circular shaft studied in case one are the same as those in Ref.^20^ ($l = 40\;{\text{in}}$, $D = 1\;{\text{in}}$, $G = 1.2 \times 10^{7} \;{\text{psi}}$, $\rho = 0.283\;{\text{lbm}}/{\text{in}}^{3}$). For convenience, non-dimensional parameters were introduced: $I_{{m,\;i}}^{*} = \rho I_{p} l/J_{{m,\;i}}$, $\xi = x/l$, $\xi _{i} = x_{{m,\;i}} /l$, $\beta = \omega /l$.

Substituting $I_{m,\;1}^{*}$, $\xi$, $\xi_{1}$ into Eq. (29). Then non-dimensional frequency coefficients can be obtained. When $\xi_{1} = 1$, the Eigen equation is given as Eq. (31), which is the same as it in Ref.^6^.

31 $$\beta \tan \beta = I_{m,1}^{*} .$$

When $\xi_{1} = 0.5$, the Eigen equation is given as

32 $$\beta \tan \beta = 2I_{m,1}^{*} .$$

In order to compare the results of AM with the corresponding ones obtained by Gorman^16^ and Chen^20^, the lowest five non-dimensional frequency coefficients, $\beta_{j}$ (j = 1–5) were shown in Table 1. Table 1 shows that the results of AM, Gorman^16^, and Chen^20^ are in good agreement, which means that the proposed method is accurate and effective for a simple model.

**Table 1:** The lowest five non-dimensional frequency coefficients for a cantilever shaft carrying a single rotary inertia.

$I_{m,\;1}^{*} = \frac{{\rho I_{p} l}}{{J_{m,1} }}$	Methods	Non-dimensional frequency coefficients				
		$\beta_{1}$	$\beta_{2}$	$\beta_{3}$	$\beta_{4}$	$\beta_{5}$
**(a) Located at **$\user2{\xi }_{1} = \user2{x}_{{\user2{m},{\mathbf{1}}}} /\user2{l} = {\mathbf{1.0}}$						
0.1	Chen^20^	0.311053	3.173097	6.299059	9.435376	12.574323
	Gorman^16^	0.311050	3.173098	6.299058	9.435379	12.574318
	AM	0.311053	3.173097	6.299059	9.435376	12.574323
1	Chen^20^	0.860333	3.425618	6.437298	9.529334	12.645287
	Gorman^16^	0.860336	3.425617	6.437293	9.529336	12.645287
	AM	0.860334	3.425618	6.437298	9.529334	12.645287
10	Chen^20^	1.428870	4.305802	7.228110	10.200262	13.214185
	Gorman^16^	1.428871	4.305799	7.228112	10.200260	13.214186
	AM	1.428870	4.305801	7.228110	10.200263	13.214186
**(b) Located at **$\user2{\xi }_{1} = \user2{x}_{{\user2{m},{\mathbf{1}}}} /\user2{l} = {\mathbf{0.5}}$						
0.1	Chen^20^	0.432841	3.203935	6.314846	9.445948	12.582264
	Gorman^16^	0.432837	3.203938	6.314849	9.445946	12.582268
	AM	0.432841	3.203935	6.314846	9.445948	12.582265
1	Chen^20^	1.076874	3.643597	6.578333	9.629560	12.722299
	Gorman^16^	1.076869	3.643596	6.578332	9.629563	12.722300
	AM	1.076874	3.643597	6.578334	9.629560	12.722299
10	Chen^20^	1.496129	4.491480	7.495412	10.511670	13.541976
	Gorman^16^	1.496127	4.491478	7.495413	10.511674	13.541977
	AM	1.496129	4.491480	7.495412	10.511670	13.541977

### Case two

**Figure 5:**
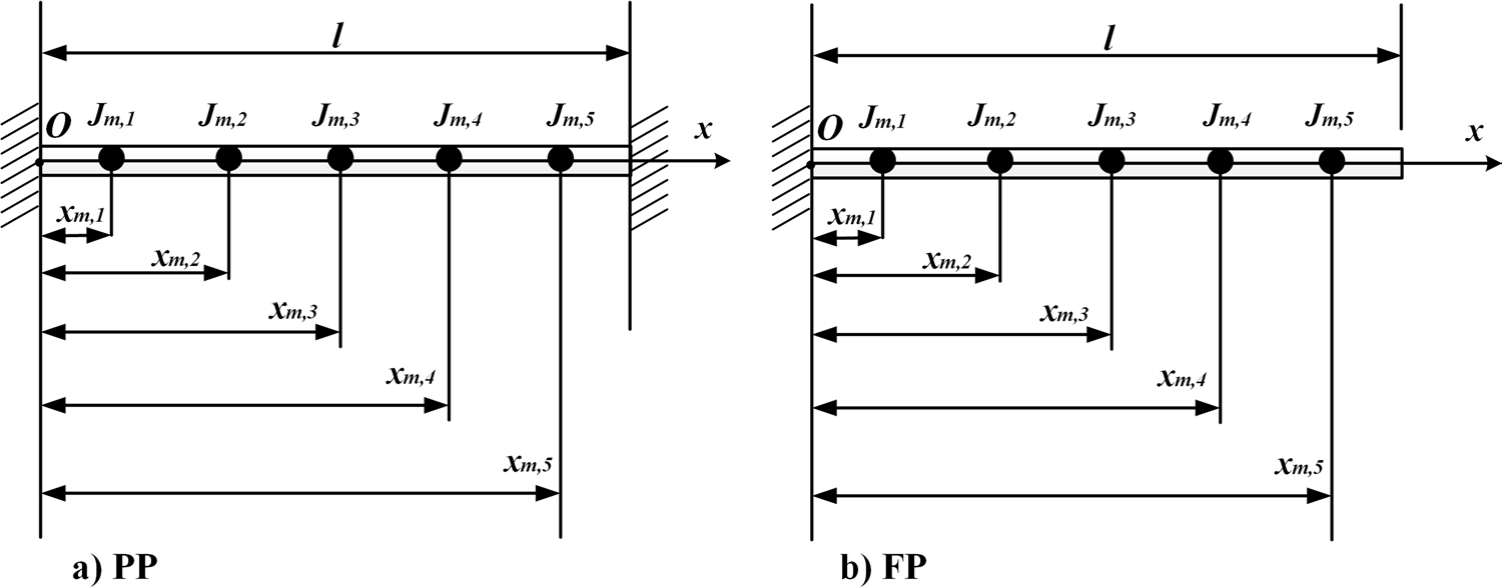
The shaft carrying five rotary inertias.

**Figure 6:**
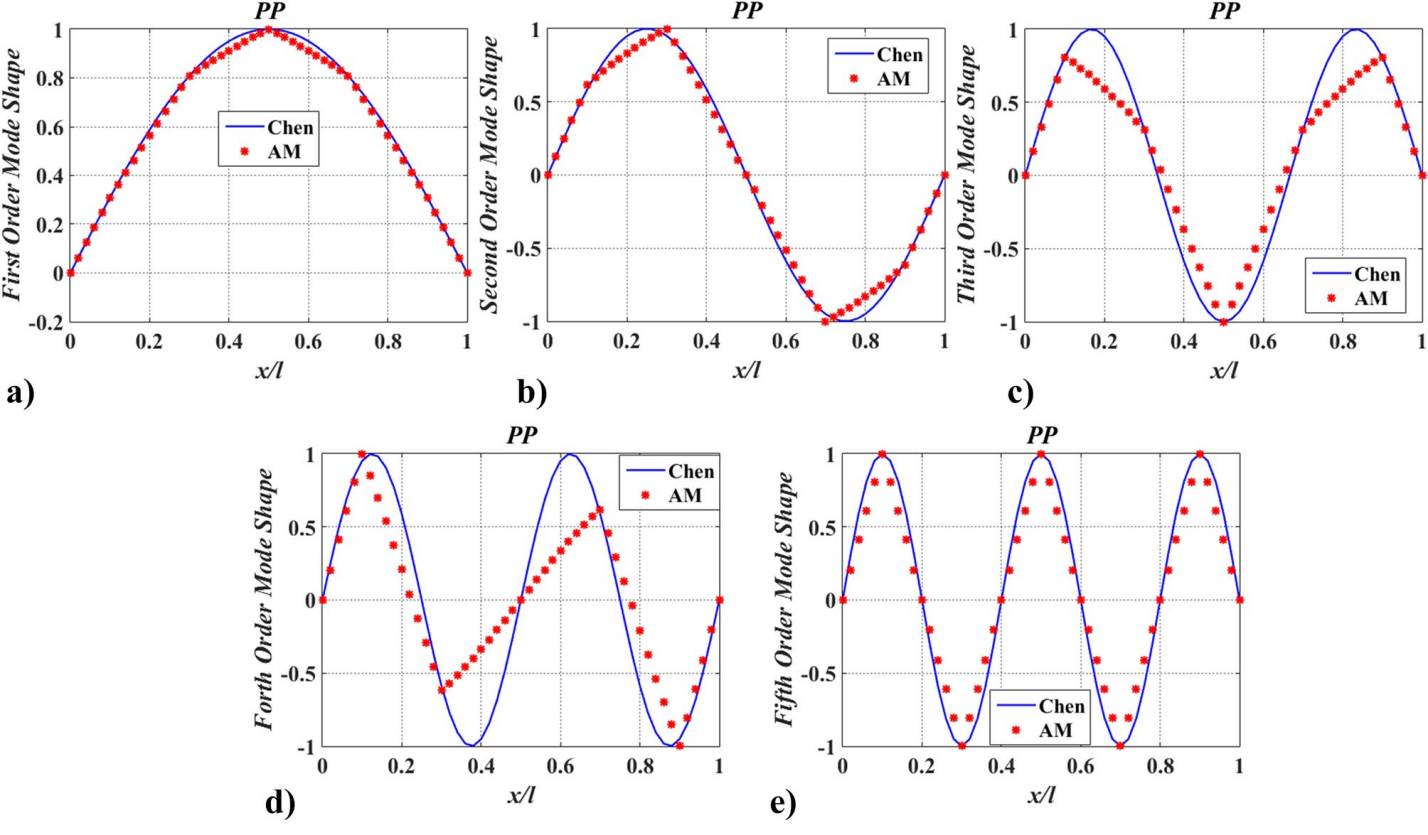
The lowest five mode shapes of twisting angles for the shaft carrying five concentrated elements in the PP support condition obtained from Chen^20^ and AM (This figure was created via Matlab 2015, https://ww2.mathworks.cn/products.html?s_tid=gn_ps, sub-images in Figs. 6, 7, 13, 14, 16, 17, 18, 19, 20 were also created by Matlab 2015).

**Figure 7:**
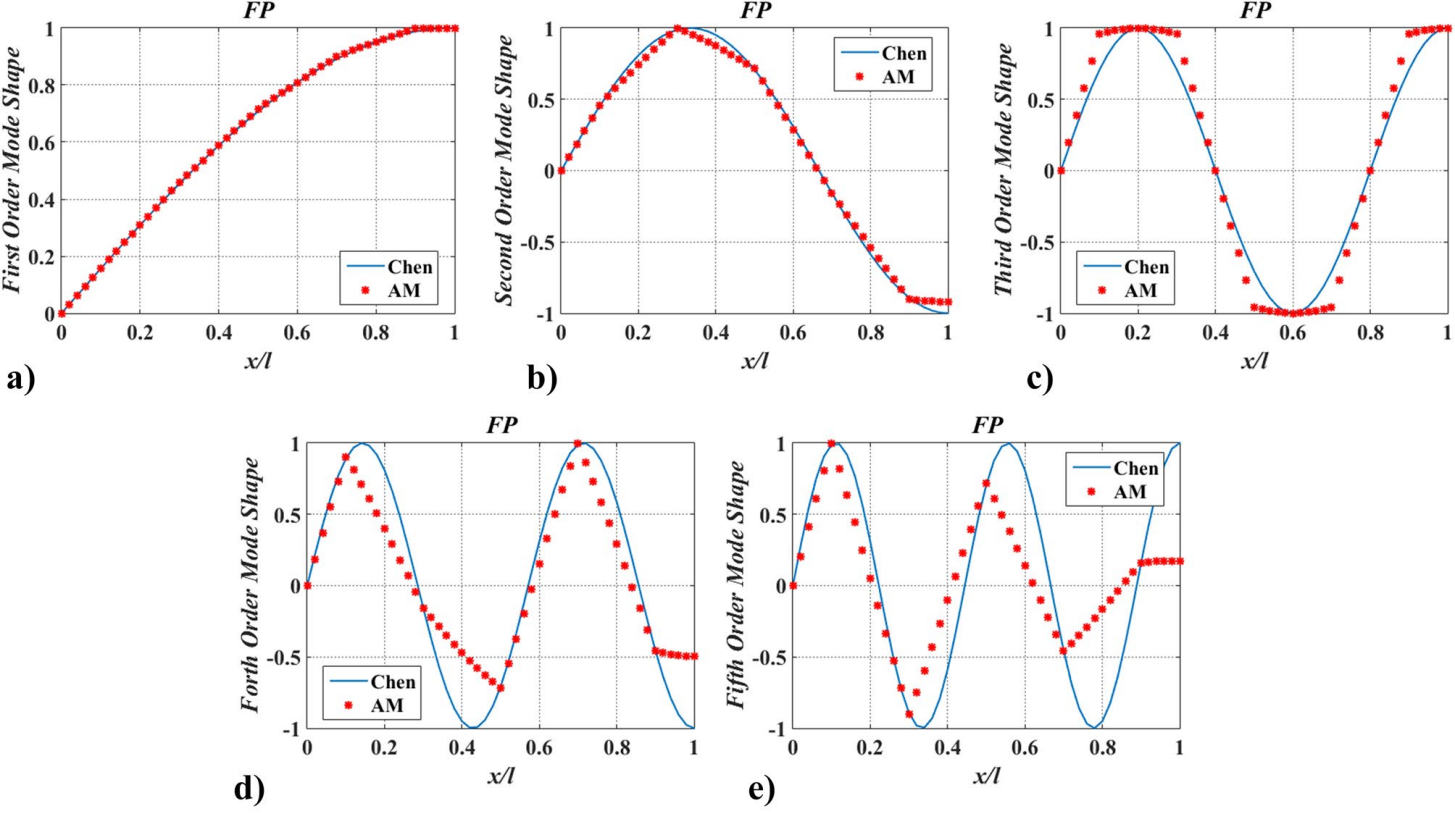
The lowest five mode shapes of twisting angles for the shaft carrying five concentrated elements in the FP support conditions obtained from Chen^20^ and AM.

Further, to verify the applicability of the proposed method in complex models, case two was adopted. Hereby, a shaft carrying five inertias, as shown in Fig. 5 was studied. The dimensions and physical properties of the shaft are the same as in case one. Locations of rotary inertia are $\xi_{1} = 0.1$, $\xi_{2} = 0.3$, $\xi_{3} = 0.5$, $\xi_{4} = 0.7$, and $\xi_{5} = 0.9$. Magnitudes of rotary inertia are $I_{m,\;i}^{*} = 1.0$ ($i = 1{-}5$). Meanwhile, boundary conditions of PP and FP were considered. The lowest five natural frequencies, $\Omega_{j}$ ($j = 1 - 5$), of the shaft were compared with FEM and Chen^20^, as shown in Table 2, and the corresponding mode shapes of twisting angles were shown in Figs. 6 and 7.

**Table 2:** The lowest five natural frequencies for the shaft carrying five rotary inertia.

Boundary conditions	Methods	Natural frequencies (rad/s), $\Omega = \sqrt \lambda$				
		$\Omega_{1}$	$\Omega_{2}$	$\Omega_{3}$	$\Omega_{4}$	$\Omega_{5}$
PP	AM	206.38630	397.88839	557.56440	665.88757	704.63711
	Chen^20^	206.38625	397.88841	557.56443	665.88763	704.63713
	$\varepsilon$(× 10^–8^)	− 0.24	4.01	5.70	8.36	2.16
	FEM	206.36478	397.84384	557.43061	665.69385	704.36091
	$\varepsilon$(× 10^–4^)	− 1.04	− 1.12	− 2.40	− 2.91	− 3.92
FP	Chen	104.09671	304.98930	482.88005	619.40413	694.81099
	Chen^20^	104.09671	304.98929	482.88004	619.40419	694.81096
	$\varepsilon$(× 10^–8^)	− 1.47	− 1.97	− 2.45	9.41	− 4.14
	FEM	104.07651	304.92280	482.77525	619.19421	694.57263
	$\varepsilon$(× 10^–4^)	− 1.94	− 2.18	− 2.17	− 3.39	− 3.43

The differences between $\Omega_{AM}$ and $\Omega_{{{\text{Re}} {\text{ference}}}} \;(\Omega_{FEM} ,\Omega_{Chen} )$ shown in the parentheses ( ) of Table 2 were calculated with the formula: $\varepsilon _{j} = (\Omega _{{\text{Re} {\text{ference, }}j}} - \Omega _{{AM{\text{, }}j}} )/\Omega _{{AM{{, }}j}}$, where $\Omega_{FEM,j}$ and $\Omega_{Chen,j}$ denote the j-th natural frequencies of the shafts carrying ‘‘five’’ torsional springs obtained from AM, Chen^20^ and FEM, respectively. From Table 2 one finds that values of $\Omega_{{AM{, }j}}$ agreement with $\Omega_{FEM,j}$ and $\Omega_{Chen,j}$ (for PP and FP shafts), hence the accuracy of the AM is good. It also can be found that values of $\varepsilon_{j}$ between $\Omega_{Chen,j}$ and $\Omega_{AM}$ are all smaller than those between $\Omega_{FEM,j}$ and $\Omega_{AM}$. This is because the FEM model is approximate, and the calculation accuracy of natural frequency can be improved by improving the degree of freedom of the FEM model.

**Figure 8:**
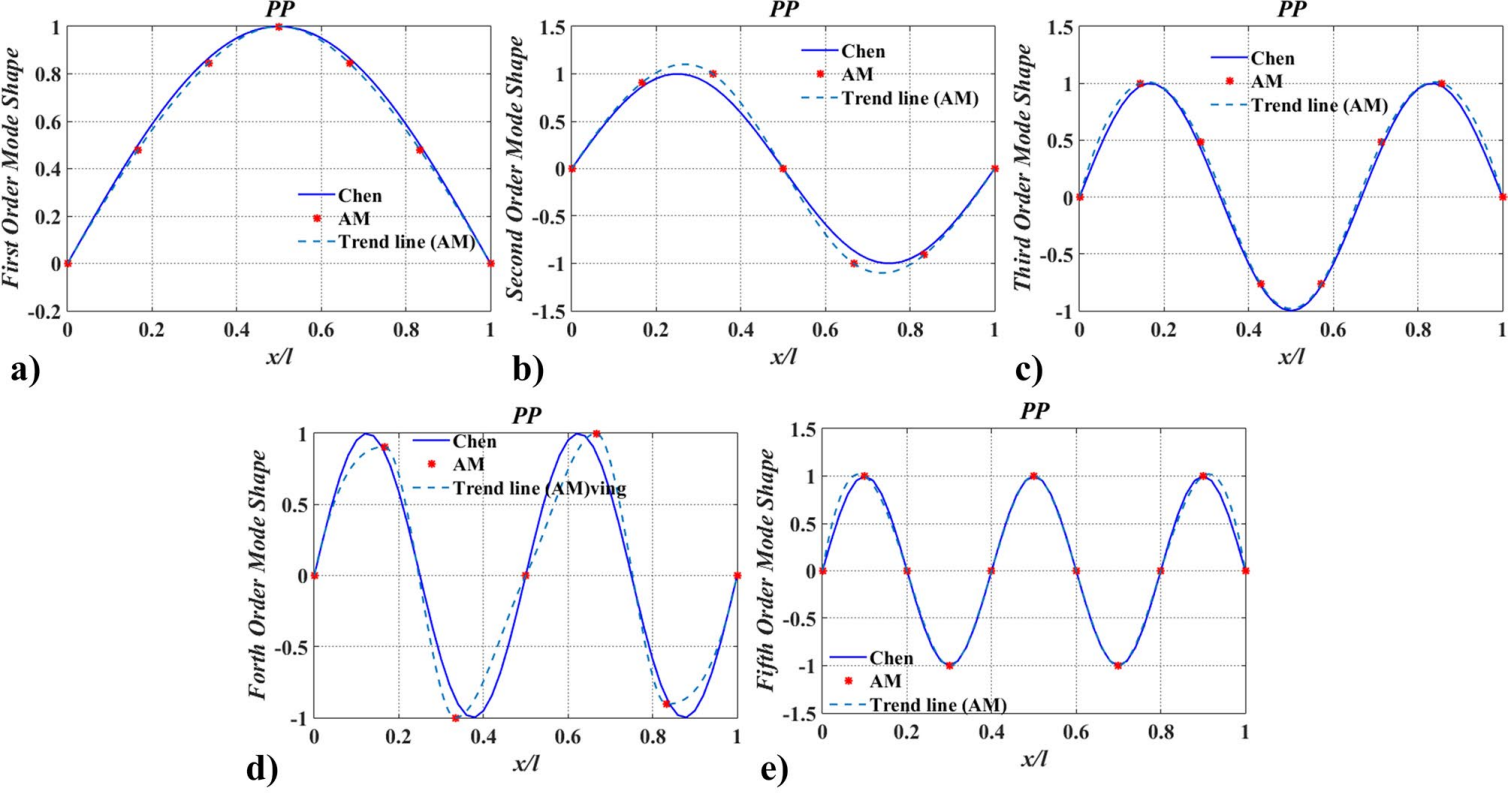
The lowest five mode shapes of twisting angles for the shaft carrying five concentrated elements in the PP support conditions obtained from Chen^20^ and AM (small number of discrete points).

**Figure 9:**
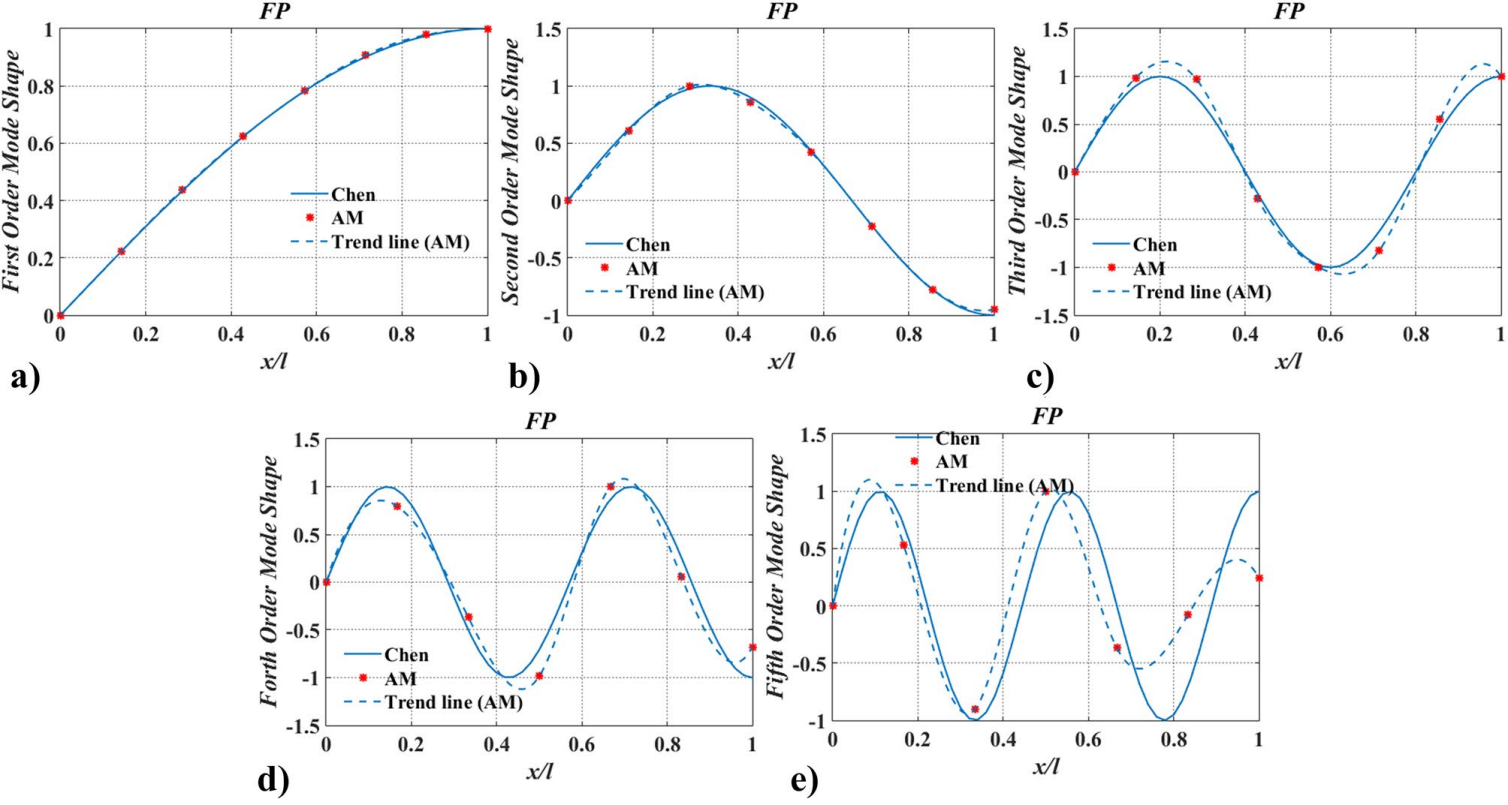
The lowest five mode shapes of twisting angles for the shaft carrying five concentrated elements in the FP support conditions obtained from Chen^20^ and AM (small number of discrete points).

Figures 6 and 7 show the lowest five mode shapes of twisting angles for the shaft carrying five concentrated elements in the support conditions: PP and PF, obtained from AM and Chen^20^. Mode shapes’ variation characteristics with $x/l$, obtained by AM, are almost identical to the ones calculated by Chen^20^. However, for the same order mode, the mode shape obtained via AM is slightly different from Chen’s at the points where rotary inertias exist, which should be blamed on the application of the conclusion from Chen^20^ and the way of mapping. In Ref.^20^ it was pointed out that mode shapes of the shafts carrying five inertias are almost coincident with the ones for the shaft without carrying any concentrated elements and the former was not shown. To compare the results of the mode shapes, the aforementioned conclusion was directly used in this paper. The mapping way of mode shape was combing limited discrete points with trend lines. As a result, if the number of discrete points is small, a similar conclusion to Ref.^20^ can be obtained via AM, as shown in Figs. 8 and 9. Once the number of discrete points is increased, the aforementioned conclusion is no longer accurate (shown in Figs. 6, 7). In fact, concentrated elements have a significant influence on mode shapes, which can be found in many pieces of literatures^7,21,25–27^.

**Figure 10:**
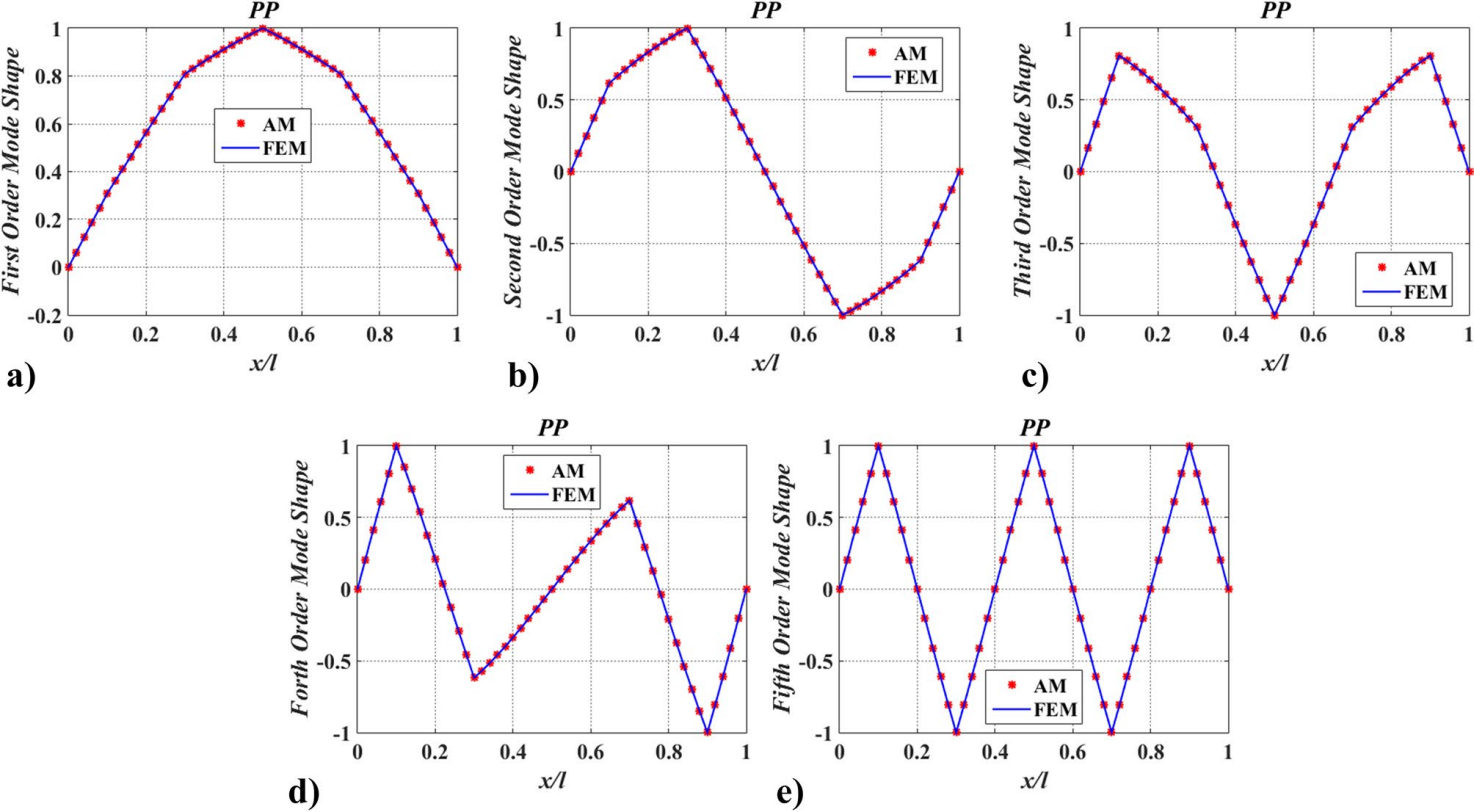
The lowest five mode shapes of twisting angles for the shaft carrying five concentrated elements in the PP support conditions obtained from FEM and AM (small number of discrete points).

**Figure 11:**
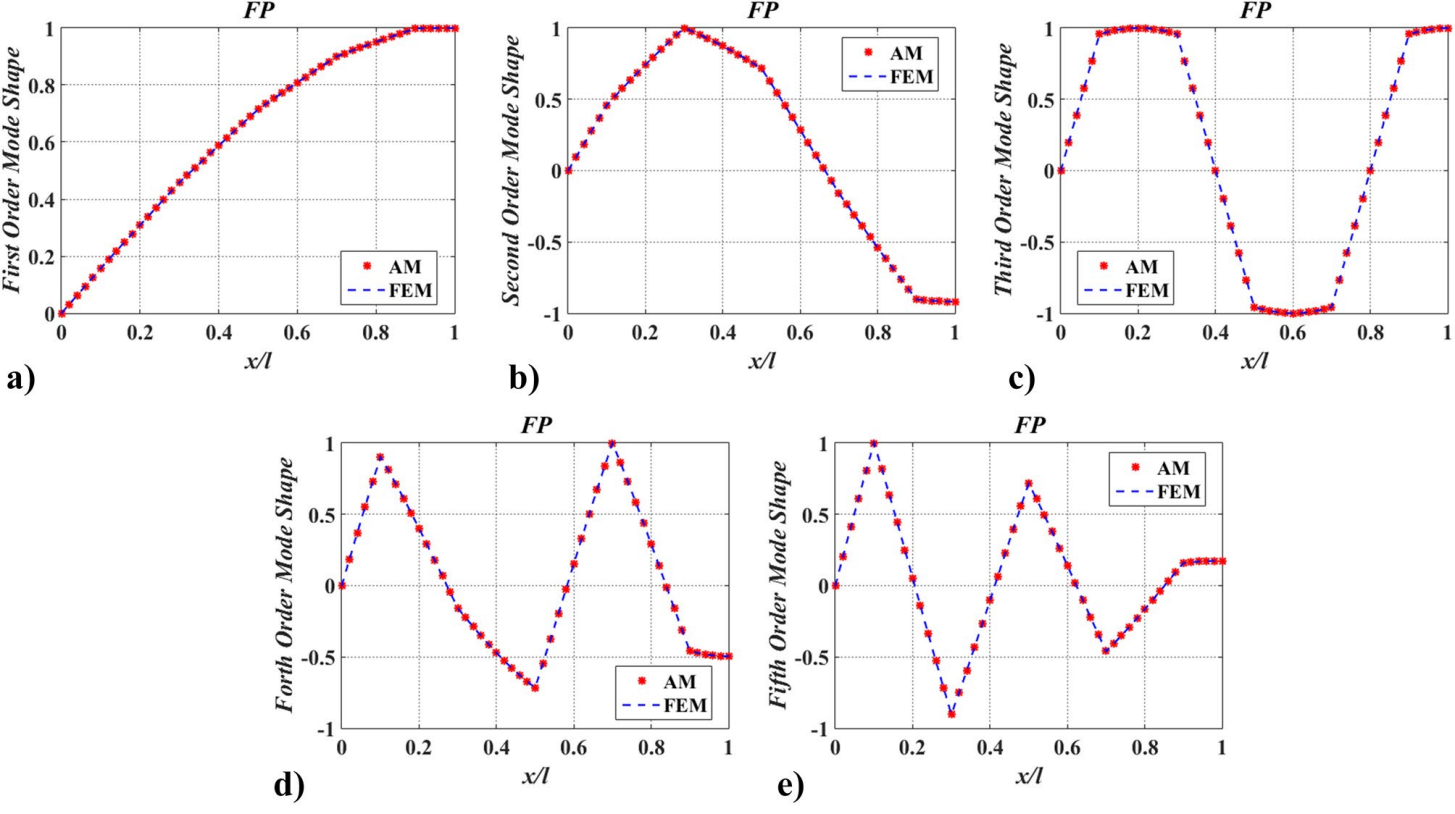
The lowest five mode shapes of twisting angles for the shaft carrying five concentrated elements in the FP support conditions obtained from FEM and AM (small number of discrete points).

To verify the accuracy of the mode shapes obtained via the presented method, FEM was employed (solved by Ansys 14.0 https://www.ansys.com/zh-cn/products/structures/ansys-mechanical). The comparison of the lowest five mode shapes obtained by FEM with 51-element uniform discrete and AM was given in Figs. 10 and 11. While modeling by FEM, a simplified model of a uniform discrete section should include a sufficient number of elements with higher-order interpolation functions. Here, beam188 with quadric shape functions for 3-D (3-node) line element was applied. From Figs. 10 and 11, it can be seen that AM results are in good agreement with FEM results, indicating that the function of the mode shape via the proposed method is accurate.

The findings in Table 2 and Figs. 6, 7, 8, 9, 10 and 11 indicate that the proposed method is accurate and effective for complex models. The results also show that concentrated elements have a significant influence on the modal shape. This phenomenon has also been found in Ref.^7,21,25–27^, which is another evidence of the accuracy of AM. The reason for the phenomenon is given in “Explanation of Phenomenon 5” section.

### Case three

**Figure 12:**
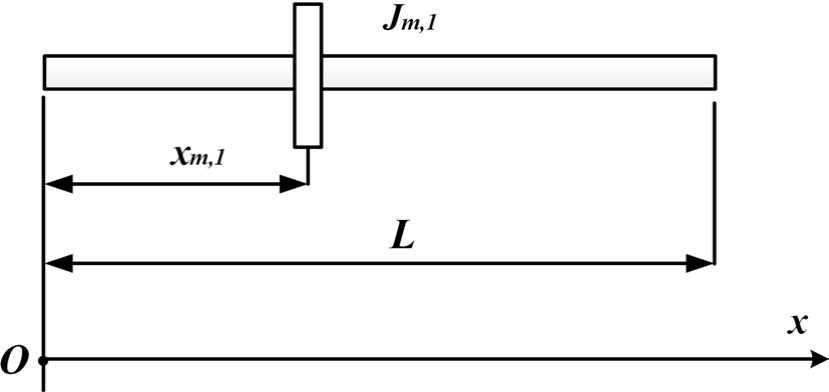
The FF shaft with one moving concentrated inertia.

**Figure 13:**
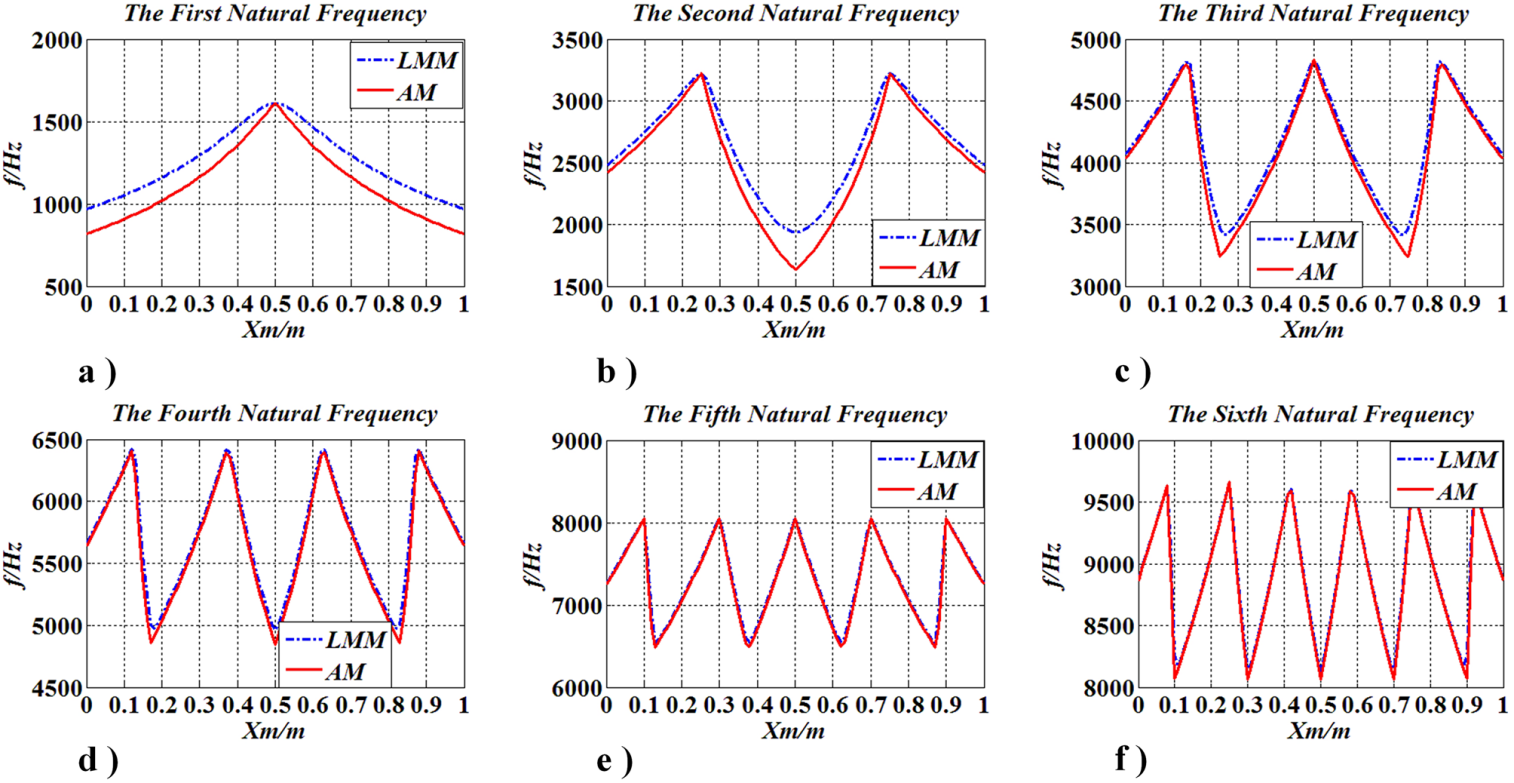
The lowest six natural frequencies of the FF shaft carrying one moving concentrated inertia.

To determine the engineering applicability of the method, case three was employed. A free boundary shaft with one moving concentrated inertia, as shown in Fig. 12, was studied. The value of parameters are: $N_{m} = 1$, $l = L = 1\;{\text{m}}$, $D = 0.1\;{\text{m}}$, $H_{m,1} = 0.1\;{\text{m}}$, $R_{m,1} = 0.2\;{\text{m}}$, $\rho = 7850\;{\text{kg}}/{\text{m}}^{3}$, $G = 81.5 \times 10^{9} \;{\text{Pa}}$, $x_{m,\;1} = x_{m}$, $J_{m,1} = J_{m}$. The results were compared with the lumped mass method (LMM) shown in Fig. 13.

From Fig. 13, it is seen that the lowest six natural frequencies of the FF shaft carrying one concentrated inertia analyzed by AM are the same as those obtained by LMM, which suggests that the presented method is applicable in practice.

According to the aforementioned comparisons, it is believed that the AM is an effective way for the title problem.

## Application of AM

In order to find the variable characteristics of natural frequencies with the change of concentrated inertias and boundary conditions to guide the torsional vibration design of shafting, this section is introduced. The dimensions and physical properties of the circular shaft are the same as in case three.

### Position influence of the concentrated inertia

The model of case three was employed in this subsection. The position of the single concentrated inertia $J_{m}$ is changed from $x_{m} = 0$ to $x_{m} = l$. The lowest six natural frequencies are shown in Fig. 13, which shows that (I) the value of the first natural frequency $f_{N} (N = 1)$ ($k$ is the order of the natural frequency) increases when $x_{m}$ changes from $x_{m} = 0$ to $x_{m} = {l \mathord{\left/ {\vphantom {l 2}} \right. \kern-\nulldelimiterspace} 2}$, whereas it reduces within ${l \mathord{\left/ {\vphantom {l 2}} \right. \kern-\nulldelimiterspace} 2} \le x_{m} \le l$, the maximum of the first natural frequency $f_{\max ,\;N,\;k} (N = 1,\;k = 1)$ is at $x_{m} = {l \mathord{\left/ {\vphantom {l 2}} \right. \kern-\nulldelimiterspace} 2}$ ($k$ is the number of the maximum natural frequency); (II) The value of the second natural frequency $f_{N} (N = 2)$ increases in two intervals ($0 \le x_{m} \le {l \mathord{\left/ {\vphantom {l 4}} \right. \kern-\nulldelimiterspace} 4}$, ${l \mathord{\left/ {\vphantom {l 2}} \right. \kern-\nulldelimiterspace} 2} \le x_{m} \le {{3l} \mathord{\left/ {\vphantom {{3l} 4}} \right. \kern-\nulldelimiterspace} 4}$) and reduces in intervals of ${l \mathord{\left/ {\vphantom {l 4}} \right. \kern-\nulldelimiterspace} 4} \le x_{m} \le {l \mathord{\left/ {\vphantom {l 2}} \right. \kern-\nulldelimiterspace} 2}$ and ${{3l} \mathord{\left/ {\vphantom {{3l} 4}} \right. \kern-\nulldelimiterspace} 4} \le x_{m} \le l$, the second natural frequency $f_{\max ,\;N,k} (N = 2,\;k = 1 - 2)$ is maxima at $x_{m} = {l \mathord{\left/ {\vphantom {l 4}} \right. \kern-\nulldelimiterspace} 4}$ and $x_{m} = {{3l} \mathord{\left/ {\vphantom {{3l} 4}} \right. \kern-\nulldelimiterspace} 4}$, and the minimum $f_{\min ,\;N,\;k}$ $(N = 2,\;k = 1)$ is at $x_{m} = {l \mathord{\left/ {\vphantom {l 2}} \right. \kern-\nulldelimiterspace} 2}$; (III) the similar results can be found in higher natural frequencies. The findings suggest that the natural frequency of torsional vibration of shafting can be designed by changing the location of concentrated elements, but there are limits that $\min \left\{ {f_{\min ,\;N,\;k} } \right\}$ ≤ $f_{k}$ ≤ $\max \left\{ {f_{\max ,\;N,\;k} } \right\}$.

### Value influence of the concentrated inertia

**Figure 14:**
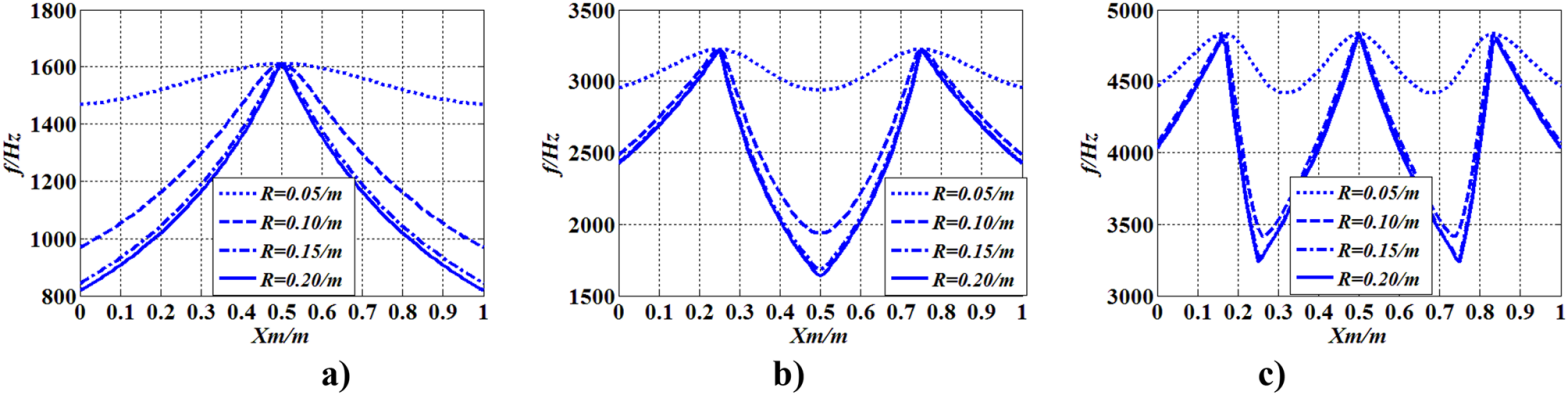
Changes of frequencies with $R\left( {J_{m} } \right)$ and $x_{m}$.

On the basis of “Position influence of the concentrated inertia” section, the value of the single concentrated inertia $J_{m}$ with changing radius $R\;\left( {R = 0.05\;{\text{m}},\;0.10\;{\text{m}},\;0.15\;{\text{m}},\;0.20\;{\text{m}}} \right)$ was investigated, as shown in Fig. 14.

In Fig. 14, when the magnitude of the concentrated inertia $R\left( {J_{m} } \right)$ is constant, as $x_{m}$ changes, variation characteristics of natural frequencies with $x_{m}$ are the same as conclusions in “Position influence of the concentrated inertia” section. When $x_{m}$ is constant, as $R\left( {J_{m} } \right)$ increases, natural frequencies increases with $R\left( {J_{m} } \right)$. However, $f_{\max ,\;k,n}$ is not affected by $R\left( {J_{m} } \right)$, whereas $f_{\min ,\;k,n}$ increases with $R\left( {J_{m} } \right)$. The results indicate that the natural frequency of torsional vibration of shafting can be designed via changing $R\left( {J_{m} } \right)$ except $f_{\max ,\;k,n}$.

### The concentrated inertia located at a certain point

**Figure 15:**
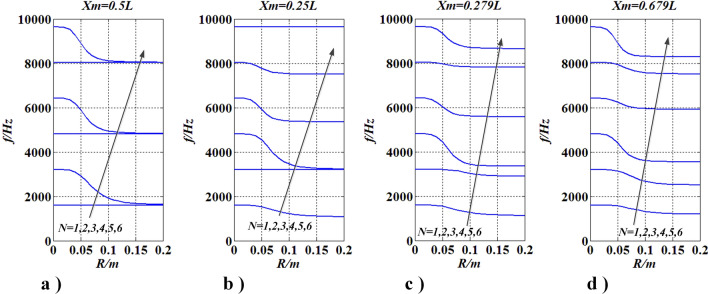
Changes of frequencies with $R\left( {J_{m} } \right)$ and $x_{m} = {\text{constant}}$ (This figure was created via Visio 2013 and Matlab 2015, Figs. 22, 23 were created by them too).

**Figure 16:**
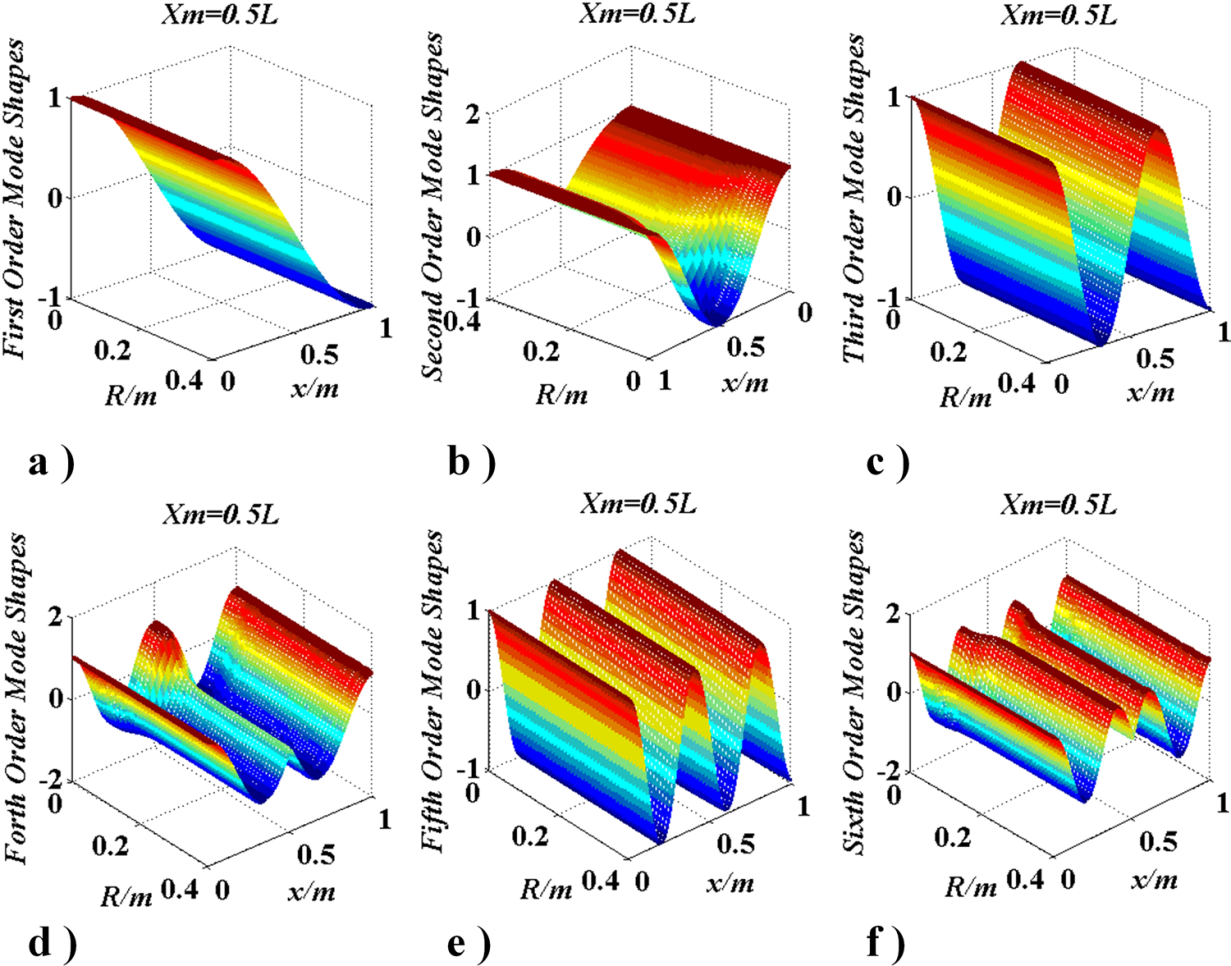
Curves of modes change with $R\left( {J_{m} } \right)$ and $x_{m} = {l \mathord{\left/ {\vphantom {l 2}} \right. \kern-\nulldelimiterspace} 2}$.

**Figure 17:**
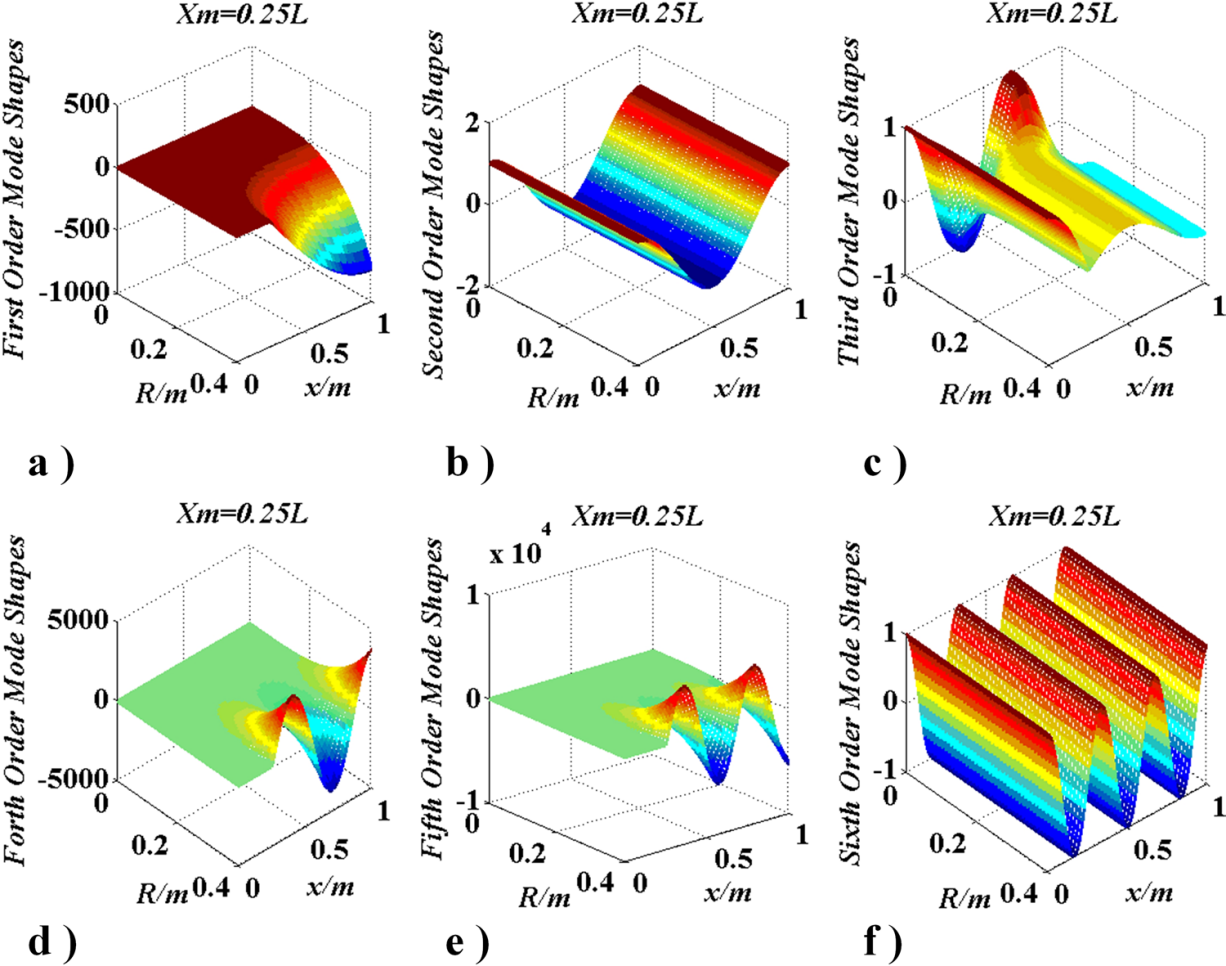
Curves of modes change with $R\left( {J_{m} } \right)$ and $x_{m} = {l \mathord{\left/ {\vphantom {l 4}} \right. \kern-\nulldelimiterspace} 4}$.

To further investigate the phenomenon in “Position influence of the concentrated inertia” and “Value influence of the concentrated inertia” sections, the model in case three continued to be studied. In this case, $J_{m}$ has a certain position ($x_{m} = 0.5l,0.25l,0.279l,$ and $0.679l$) and a changing radius $R\left( {0 \le R \le 0.2m} \right)$. Natural frequencies (1st–6th) were given in Fig. 15, and mode shapes were given in Figs. 16 and 17.

From Fig. 15, when $J_{m}$ is located at $x_{m} = l/n_{1} (n_{1} = 2,4,6, \ldots )$, the $n_{1} (2q + 1)/2\;(q = 0,1,2, \ldots )$-th natural frequency does not change with $R\left( {J_{m} } \right)$, the other reduce with $R\left( {J_{m} } \right)$, while each frequency trends to a certain value and the $n_{1} (2q + 1)/2 + 1$-th natural frequency equals to the $n_{1} (2q + 1)/2$-th natural frequency if $R\left( {J_{m} } \right) \to \infty$; when $J_{m}$ is not located at $x_{m} = l/{n_1}\, (n_{1} = 2,4,6, \ldots )$, each natural frequency reduces with $R\left( {J_{m} } \right)$ and each frequency trends to a certain value if $R\left( {J_{m} } \right) \to \infty$. The mentioned phenomena also influence the mode shapes of the shaft. Therefore, Figs. 16 and 17 were given for further discussion.

In Fig. 16, mode shapes of the lowest six natural frequencies were given when $J_{m}$ is located at $x_{m} = l/2$. It can be seen that the ${{n_{1}} (2q + 1)}/2\;(n_{1} = 2,\;q = 0,1,2, \ldots )$-th order mode shape did not change with $R$, the other mode shapes change evidently and the range of those change mode shapes are beyond the interval [− 1, 1]^28–30^. From Fig. 17 the same conclusions can be obtained.

### Number influence of the concentrated inertias

**Figure 18:**
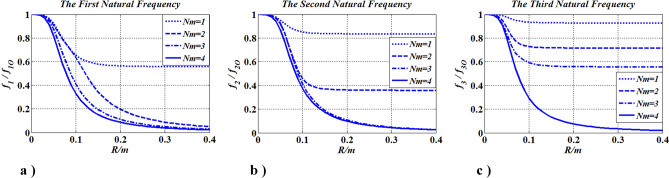
Frequencies change with $R\left( {J_{m} } \right)$ and $N_{m}$.

Number influence of concentrated inertias on the natural frequency was investigated in this case (Table 3). The results were given in Fig. 18. $N_{m} = 1{-}4$, $0 \le R \le 0.4\;{\text{m}}$, $f_{j} \left( {j = 1,2,3, \ldots } \right)$: the $j$-th natural frequency and $f_{j\;O}$: the $j$-th natural frequency, when $R = 0$.

**Table 3:** Distribution of concentrated inertias.

Number $N_{m}$	Position $x_{m,\;i}$	Magnitude $R_{m,i}$	Boundary conditions
1	$x_{m,1} = 0.1l$	$R$	FF
2	$x_{m,1} = 0.1l$, $x_{m,2} = 0.3l$	$R$	FF
3	$x_{m,1} = 0.1l$, $x_{m,2} = 0.3l$, $x_{m,3} = 0.7l$	$R$	FF
4	$x_{m,1} = 0.1l$, $x_{m,2} = 0.3l$, $x_{m,3} = 0.7l$, $x_{m,4} = 0.9l$	$R$	FF

Figure 18 shows that each natural frequency reduces when values of the concentrated inertias increase or when the number of concentrated inertias increases. These findings indicate that the natural frequency can be controlled by changing the magnitude and number of inertia.

### The influence of boundary conditions

**Figure 19:**
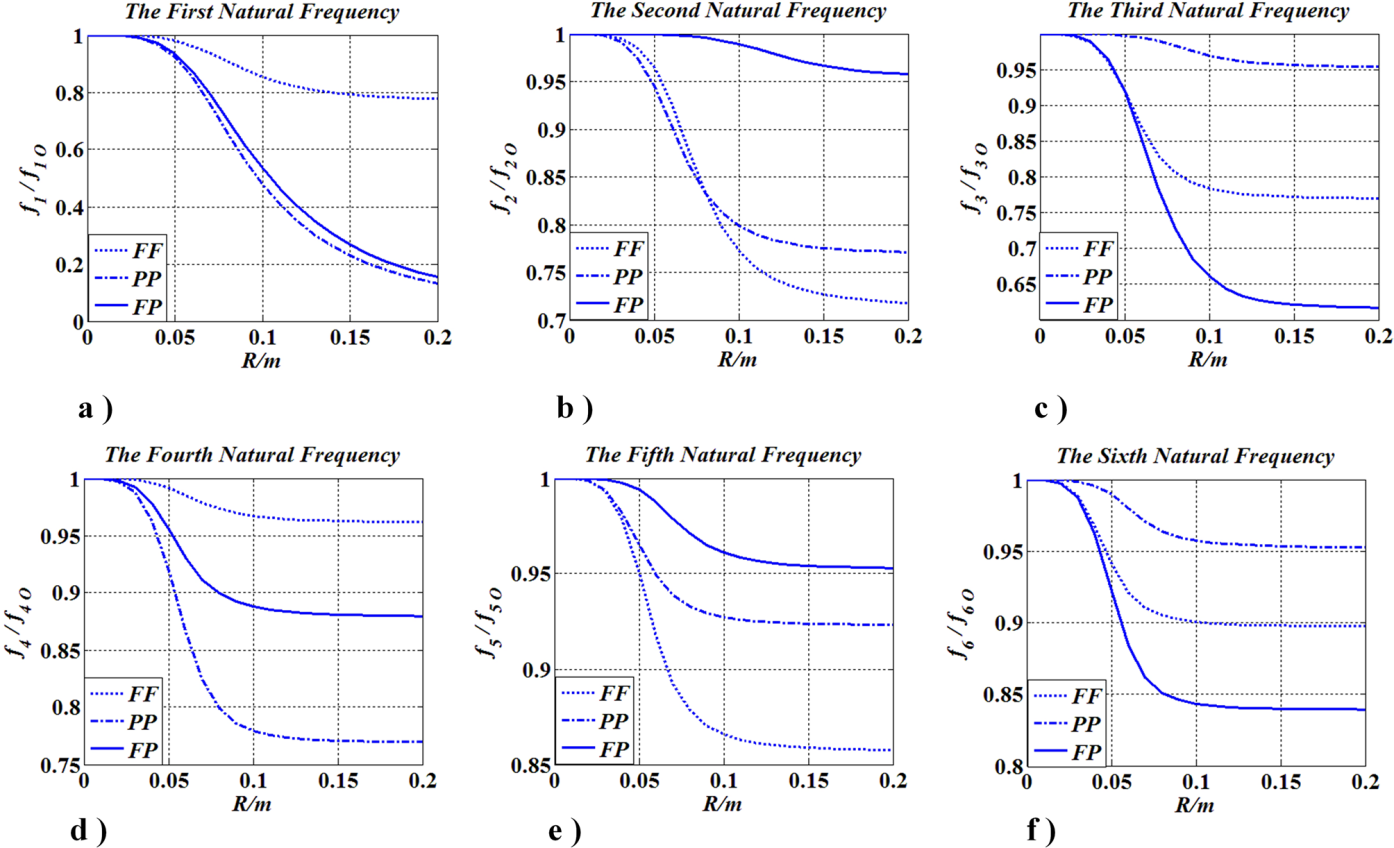
Frequencies change with $R\left( {J_{m} } \right)$ and boundary conditions.

**Figure 20:**
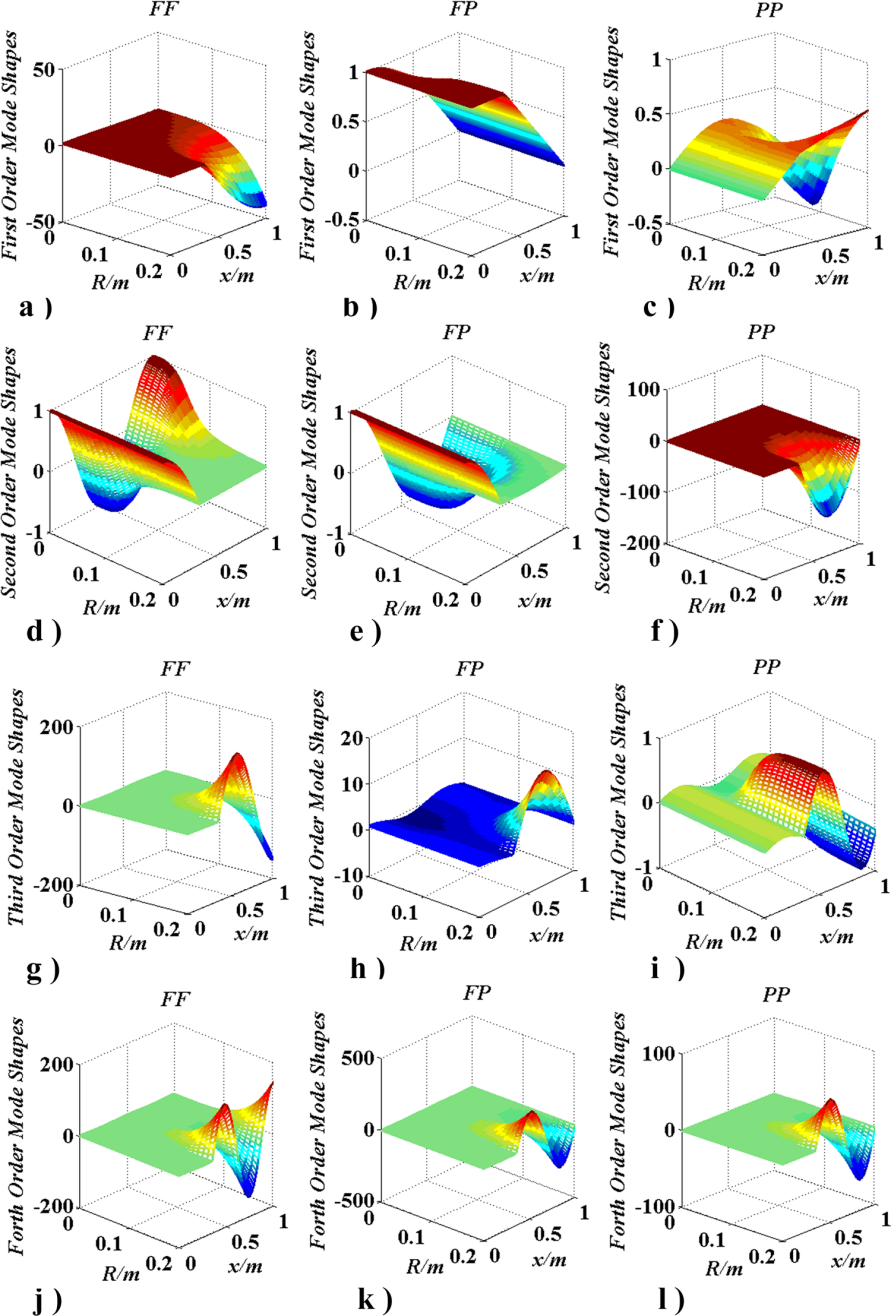
Changes of mode shapes with $R\left( {J_{m} } \right)$ and boundary condition.

In this case, boundary conditions (FF, PP, and FP) influence on natural frequencies was studied (as shown in Figs. 19, 20). $N_{m} = 1$, $0 \le R \le 0.2m$, $x_{m,1} = x_{m} = 0.35l$, $f_{j} \left( {j = 1,2,3, \ldots } \right)$ is the $j$-th natural frequency, and $f_{j\;O}$ is the $j$-th natural frequency when $R = 0\left( {J_{m} = 0} \right)$.

Figure 19 shows that, regardless of the boundary conditions, each natural frequency becomes smaller as the radius of the concentrated inertia becomes larger, which suggests that the conclusions obtained in “Position influence of the concentrated inertia”, “Value influence of the concentrated inertia”, “The concentrated inertia located at a certain point” and “Number influence of the concentrated inertias” sections are not affected by boundary conditions. In Fig. 20, it can be found that when $J_{m}$ is not located at $x_{m} { = 0}{\text{.35}}l$, every mode shape, regardless of the boundary conditions, is changed with the increase of $J_{m}$, the range of every modal is beyond the interval [− 1,1]. The findings means that the mode shape will be affected by the magnitude of the concentrated inertia.

## Discussion of some phenomena

In “Application of AM” section, five interesting phenomena were observed. Phenomenon 1: When the concentrated inertia is located at $x_{m} = {l \mathord{\left/ {\vphantom {l {n_{1} }}} \right. \kern-\nulldelimiterspace} {n_{1} }}(n_{1} = 2,4,6, \ldots )$, some natural frequencies do not change with the magnitude of concentrated inertias; Phenomenon 2: As $R\left( {J_{m} } \right) \to \infty$, each natural frequency tends to a certain constant; Phenomenon 3: When the concentrated inertia is located at $x_{m} = l/{n}_{1}\,(n_{1} = 2,4,6, \ldots )$ and $R\left( {J_{m} } \right) \to \infty$, the ${{n_{1}} (2q + 1)}/2 + 1$-th natural frequency equals to the ${{n_{1}} (2q + 1)}/2$-th natural frequency; Phenomenon 4: $f_{\max ,\;k,n}$, $f_{\min ,\;k,n}$ in “Position influence of the concentrated inertia” and “Value influence of the concentrated inertia” sections appear alternately when the position of $J_{m}$ is changed, the magnitude and position of $f_{\max ,\;k,n}$ are constant, but the magnitude and position of $f_{\min ,\;k,n}$ changes with $J_{m}$. Phenomenon 5: Some mode shapes are beyond the interval [− 1, 1].

### Explanation of Phenomenon 1

Phenomenon 1 is caused by the eigenvalue equation. Considering $N_{m} = 1$, and $J_{m,1} = J_{m}$, eigenfunction Eq. (25) can be expressed as

33 $$\omega \left[ {\sin \omega l{ + }\frac{\omega }{{\rho I_{p} }}J_{m} \cos \omega x_{m} \cos \omega (l - x_{m} )} \right] = 0.$$

Therefore

34 $$\omega = 0,$$

or

35 $$\sin \omega l{ + }\frac{\omega }{{\rho I_{p} }}J_{m} \cos \omega x_{m} \cos \omega (l - x_{m} ) = 0.$$

Equations (36) and (37) are obtained from Eq. (35).

36 $$\left\{ \begin{array}{*{20}l} \cos \omega x_{m} \cos \omega (l - x_{m} ) = 0,\;\sin \omega l = 0 \hfill \\ \sin \omega l{ + }\frac{\omega }{{\rho I_{p} }}J_{m} \cos \omega x_{m} \cos \omega (l - x_{m} ) \ne 0 \hfill \\ \end{array} \right.,$$

37 $$\left\{ \begin{array}{*{20}l} \sin \omega l{ + }\frac{\omega }{{\rho I_{p} }}J_{m} \cos \omega x_{m} \cos \omega (l - x_{m} ) = 0 \hfill \\ \sin \omega l \ne 0,\;\cos \omega x_{m} \cos \omega (l - x_{m} ) \ne 0 \hfill \\ \end{array} \right..$$

The solution of $\sin \omega l = 0$ can be expressed as

$$U_{1} = \left\{ {\omega _{1} = \left. {\frac{{n_{1} \pi }}{l}} \right|n_{1} = 0,1,2, \ldots } \right\}.$$

If $\cos \omega x_{m} = 0$, the solution set is

$$U_{2} = \left\{ {\omega_{2} = \frac{{\left( {2n_{2} + 1} \right)\pi }}{{2x_{m} }}\left| {n_{2} = 0,1,2, \ldots } \right.} \right\}.$$

The solution has the following form when $\cos \omega (l - x_{m} ) = 0$

$$U_{3} = \left\{ {\omega _{3} = \left. {\frac{{\left( {2n_{3} + 1} \right)\pi }}{{2\left( {l - x_{m} } \right)}}} \right|n_{3} = 0,1,2, \ldots } \right\}.$$

So the solution of Eq. (36) can be expressed as

$$U_{0} = U_{1} \bigcap {\left( {U_{2} \bigcup {U_{3} } } \right).}$$

Assuming that the solution of Eq. (37) is $U_{4}$, the solution of Eq. (24) can be obtained

$$U = U_{0} \bigcup {U_{4} } .$$

$U,U_{0} ,U_{1} ,U_{2} ,U_{3} ,U_{4}$ are all orderly solution sets.

It can be inferred from Eq. (36) that if $\cos \omega x_{m} \cos \omega (l - x_{m} ) = 0$ and $\sin \omega l = 0$, the solutions of Eq. (33) are independent with $J_{m}$. That is why values of some natural frequencies don’t change with the value change of single concentrate inertia $J_{m}$ when $J_{m}$ is at $x_{m} = l/n_{1}\,(n_{1} = 2,4,6, \ldots )$.

### Explanation of Phenomenon 2

**Figure 21:**
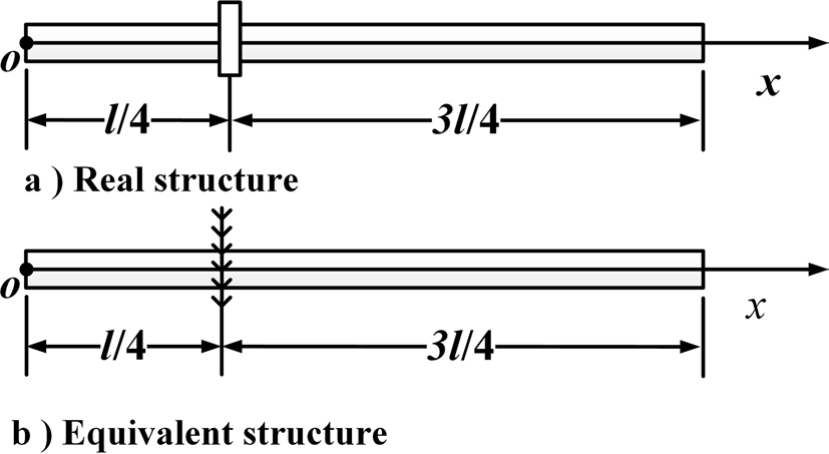
The shaft carrying single concentrated inertia $J_{m}$ located at $x_{m} = l/4$.

Phenomenon 2 is caused by boundary conditions. As $R\left( {J_{m} } \right) \to \infty$, the position where the concentrated inertia is located becomes fixed boundary conditions. To explain the reason, Fig. 21 was given.

When $R$ increases, the inertia of each concentrate inertias increases, and each of them is harder to be rotated. When $R \to \infty ,$ then $J_{m} \to \infty$, the position of each concentrate inertias is equivalent to the fixed end, and the shaft is divided into beam segments. Thus, the natural frequencies of the shaft mentioned in the title consist of the solution sets of all beam segments. Take the model in Fig. 21a for example. A shaft carrying single concentrated inertia $J_{m}$ located at $x_{m} = l/4$. When $R \to \infty$, $J_{m} \to \infty$. As a result, the equivalent structure in Fig. 21b can be obtained. When the value of each parameter is set the same as in case three, Table 4 appears. From Table 4, the conclusion is proved and this conclusion is also applicable to multi-inertia systems.

**Table 4:** Values of natural frequencies.

The order of the natural frequency	1	2	3	4	5	6
Frequencies of beam segment on the left/Hz	3222.13	9666.41	–	–	–	–
Frequencies of beam segment on the right/Hz	1074.04	3222.137	5370.228	7518.32	9666.41	–
Values of certain constants/Hz	1074.04	3222.137	3222.137	5370.23	7518.32	9666.41

### Explanation of Phenomenon 3

Phenomenon 3 has the same reason as Phenomenon 2. If $$R \to \infty$$, then $$J_{m} \to \infty$$. Thus the position of each concentrate inertias is equivalented to the fixed end. Take the model in Fig. 17b for example. Solution sets of the left part are $$\omega _{{left}} = 4\left( {r + 1/2} \right)\pi /l$$$$(r = 0,1,2, \ldots )$$, Solution sets of the right part are $$\omega _{{right}} = 4\left( {s + 1/2} \right)\pi /3l$$ $$\left( {s = 0,1,2, \ldots } \right)$$. Obviously, there are certain values of $$r$$ and $$s$$ that make $$\omega_{left} = \omega_{right}$$. Such as, $$r = 0$$ and $$s = 1$$, $$r = 1$$ and $$s = 4$$, etc. When the two sets of solutions ($$\omega_{left} ,\;\omega_{right}$$) are combined and arranged in ascending order, Phenomenon 3 appears.

### Explanation of Phenomenon 4

Maximum (minimum) values ($f_{\max ,\;k,n}$, $f_{\min ,\;k,n}$) in “Position influence of the concentrated inertia” and “Value influence of the concentrated inertia” sections appear alternately when the position of $J_{m}$ is changed. The position of each maximum value $f_{\max ,\;k,n}$ has no connection with $J_{m}$, which is caused by the reason in “Explanation of Phenomenon 1” section. If $\omega_{1} = \omega_{2}$ or $\omega_{1} = \omega_{3}$, the solution is $x_{m} = \left( {2n_{2} + 1} \right)l/2n_{1}$ or $x_{m} = \left[ {2\left( {n_{1} - n_{3} } \right) - 1} \right]l/2n_{1}$. Considering $0 \le x_{m} \le l$, $n_{1} > n_{2} \ge 0$,$n_{1} > n_{3} \ge 0$ and $n_{1} \ne 0$, the position of $f_{\max ,\;k,n}$ obtained was shown in Table 5, which is consistent with the phenomenon in “Position influence of the concentrated inertia” and “Value influence of the concentrated inertia” sections.

**Table 5:** Maximum position.

$n_{1}$	1	2	3	4	⋯
$n_{2} (n_{3} )$	0	0, 1	0, 1, 2	0, 1, 2, 3	$n_{2} (n_{3} ) = 0,1,2, \ldots n_{1}$
Maximum position $x_{m}$	$\frac{l}{2}$	$\frac{l}{4},\frac{3l}{4}$	$\frac{l}{6},\frac{l}{2},\frac{5l}{6}$	$\frac{l}{8},\frac{3l}{8},\frac{5l}{8},\frac{7l}{8}$	$\frac{l}{{n_{1} }}, \ldots ,\frac{{(2n_{1} - 1)l}}{{n_{1} }}$

**Figure 22:**
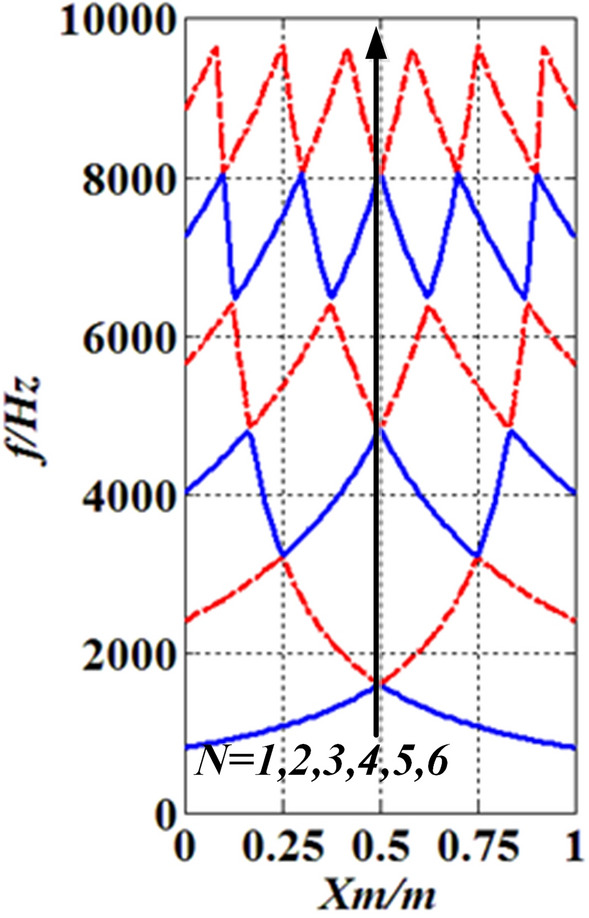
Results of AM.

**Figure 23:**
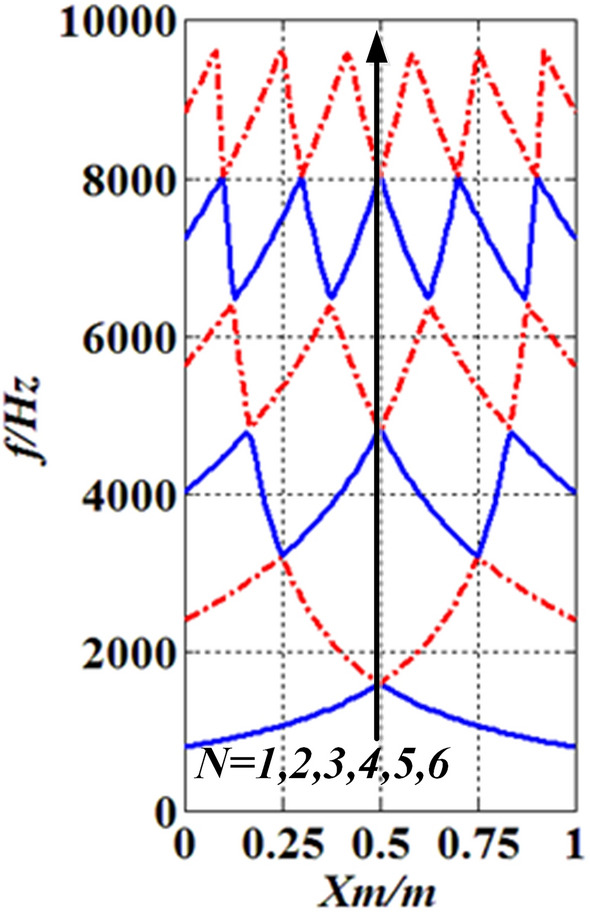
Results of LMM.

However, the magnitude and position of $f_{\min ,\;k,n}$ changes with $J_{m}$. Magnitude change of $f_{\min ,\;k,n}$ is caused by Magnitude change of $J_{m}$, which had been given in “Explanation of Phenomenon 3” section. The position change of $f_{\min ,\;k,n}$ is caused by the combination of changes in the magnitude and position of $J_{m}$. When $J_{m} \to 0$ and $x_{m}$ changes within $0 \le x_{m} \le l$, $f_{\min ,\;k,n} = f_{\max ,\;k - 1,n}$. To clearly illustrate this conclusion, Figs. 22 and 23 was given. In this case, $N_{m} = 1$, $N = k$, and values of other parameters are set the same as in case three. Figure 22 was obtained by AM, which visually reflects the relationship between $f_{\min ,\;k,n}$ and $f_{\max ,\;k - 1,n}$. In order to prove the conclusion of Fig. 22, LMM was used for calculation ($R = 1\;{\text{m}}$), and the results supported the conclusion in Fig. 22.

### Explanation of Phenomenon 5

Phenomenon 5 is caused by the magnitude change of $J_{m,i}$. As shown in Eq. (28), the mode shape $\theta (x)$ is related to $\theta (0)\cos \omega x$, $\frac{{\theta^{\prime}(0)}}{\omega }\sin \omega x$, and $\sum\limits_{i = 1}^{{N_{m} }} {\frac{{\omega J_{m,i} }}{{\rho I_{p} }}\theta (x_{m,i} )} \sin \omega (x - x_{m,i} )H(x - x_{m,i} )$. Obviously, $\frac{{\omega J_{m,i} }}{{\rho I_{p} }}\theta (x_{m,i} ) > > 1$ exists. Thus, some mode shapes are beyond the interval [− 1, 1].

## Conclusion

A novel analytical method is used to perform the torsional vibration analysis of a uniform circular shaft carrying multiple concentrated elements (rotary inertias) with arbitrary magnitudes and locations. The governing equations are obtained by the Hamiltonian principle and verified by the dynamical method. The Laplace transform is used to obtain the analytic expression of the governing equation. The correctness of the solution is verified by the comparison with the existing literature conclusions and LMM. Several general rules were obtained:

(I) The torsional vibration model established in this study is universality. The free torsional vibration of shafts, such as ship shafting, rail car shafting, submarine propulsion system shafting, and other mechanical devices, can be simulated accurately by this model.
(II) The analytical solutions of torsional vibration are given, which can be used in analyzing the natural frequencies and mode shapes of such structures and are instructive to the subsection design of shafting. The natural frequencies and modes vary with the magnitudes, positions, and the number of concentrated inertias. This provides a reference for the vibration reduction design of shafting.
(III) It has been found that the results of AM are well in agreement with those of LMM, FEM, German, and Chen. The calculation method is very simple, which may be one of the simplest tools for studying the title problem.

## Data Availability

The data used to support the findings of this study are included in the article.
